# The subcellular localization of bHLH transcription factor TCF4 is mediated by multiple nuclear localization and nuclear export signals

**DOI:** 10.1038/s41598-019-52239-w

**Published:** 2019-10-30

**Authors:** Beata Greb-Markiewicz, Wioletta Kazana, Mirosław Zarębski, Andrzej Ożyhar

**Affiliations:** 1 Department of Biochemistry, Faculty of Chemistry, Wroclaw University of Science and Technology, Wybrzeże Wyspiańskiego 27, 50-370 Wroclaw, Poland; 2 Department of Cell Biophysics, Faculty of Biochemistry, Biophysics and Biotechnology, Jagiellonian University, Gronostajowa 7, 30-387 Cracow, Poland; 3 Present Address: Ludwik Hirszfeld Institute of Immunology and Experimental Therapy, Polish Academy of Sciences, Rudolfa Weigla Street 12, 53-114 Wroclaw, Poland

**Keywords:** Mechanism of action, Post-translational modifications, Proteins, Transporters

## Abstract

Transcription factor 4 (TCF4) is a class I basic helix-loop-helix (bHLH) transcription factor which regulates the neurogenesis and specialization of cells. TCF4 also plays an important role in the development and functioning of the immune system. Additionally, TCF4 regulates the development of Sertoli cells and pontine nucleus neurons, myogenesis, melanogenesis and epithelial-mesenchymal transition. The ability of transcription factors to fulfil their function often depends on their intracellular trafficking between the nucleus and cytoplasm of the cell. The trafficking is regulated by specific sequences, i.e. the nuclear localization signal (NLS) and the nuclear export signal (NES). We performed research on the TCF4 trafficking regulating sequences by mapping and detailed characterization of motifs potentially acting as the NLS or NES. We demonstrate that the bHLH domain of TCF4 contains an NLS that overlaps two NESs. The results of *in silico* analyses show high conservation of the sequences, especially in the area of the NLS and NESs. This high conservation is not only between mouse and human TCF4, but also between TCF4 and other mammalian E proteins, indicating the importance of these sequences for the functioning of bHLH class I transcription factors.

## Introduction

Transcription factor 4 (TCF4), also known as immunoglobulin transcription factor 2 (ITF2) or SL3-3 enhancer factor 2 (SEF2), belongs to the family of ubiquitously expressed transcription factors that contain a basic helix-loop-helix (bHLH) domain. It plays an important role in a number of developmental processes^1^. There are four known mammalian representatives of class I bHLH (E proteins) transcription factors. In addition to TCF4, to this class also belong: E12 and E47 (E2a/TCF3 isoforms), and HEB (human E-box binding factor)/TCF12. The bHLH domain is responsible for homo- and heterodimer formation with other bHLH transcription factors (TFs). The region of basic amino acid residues of the bHLH domain facilitate DNA binding, indispensable for the role of transcription factors, while their function and activity is regulated by activation and repressor domains^2^. TCF4 binds the canonical enhancer-box (E-box) sequence 5′-CANNTG-3′^3^ and influences chromatin remodelling and transcription through the recruitment of p300 with histone acetyltransferases (HATs) activity^4^. TCF4 is known to exist in many isoforms, which are generated by the usage and splicing of alternative 5′ exons^5^. These isoforms differ in terms of the presence of activation and repressor domains^6^.

TCF4 expression was reported in different organs: brain, heart, kidney, lungs, muscle, spleen and testis, and in lesser amounts in the liver, prostate and ovaries^7^. It was shown that TCF4 is involved in the differentiation of plasmacytoid dendritic cells that form part of the adaptive immune system. TCF4 is also crucial for the development of lymphoid progenitor cells, giving rise to T- and B-lineage cells^8^. Additionally, TCF4 regulates the development of Sertoli cells, myogenesis, melanogenesis and epithelial-mesenchymal transition^5^.

A particularly high-level expression of TCF4 is observed in the embryonic central nervous system, mesoderm and adult brain^9^. TCF4 regulates the neurogenesis and differentiation of cells by forming heterodimers with proneuronal activators belonging to the bHLH family, like achaete-scute homolog 1 (ASCL1), protein atonal homolog 1 (ATOH1) and neurogenic differentiation factor 1 (NEUROD1). Conversely, the interaction of TCF4 with the DNA-binding protein inhibitor ID-2 (ID2) results in the formation of an inactive heterodimer, thus blocking TCF4 binding with activators^10^. To date, not much is known about the role of TCF4 in the central nervous system. However, *in situ* tests have shown that TCF4 is an important factor in the regulation of glial cell differentiation, especially the maturation of oligodendrocyte progenitors, and it also plays an important role in the regulation of the nuclei development of the pons involved in motor activity^11–13^.

Over the last several years, TCF4 has been linked to many diseases, mainly brain disorders. Genome analysis has revealed common variants in TCF4 as susceptibility loci for schizophrenia. This disease was first linked to the *TCF4* gene by Stefansson^14^, who reported a single-nucleotide polymorphisms associated with this gene. Later, studies of Chinese patients^15^ confirmed this relationship by finding additional polymorphisms. Another disease with characteristics similar to schizophrenia is bipolar disorder, which is characterized by alternating episodes of depression and mania. Performed studies revealed a decreased expression level of TCF4 in the case of both these disorders^8^. In 2007, three independent research groups revealed that the *TCF4* gene was linked to the presence of the mental disorder Pitt-Hopkins Syndrome (PHS). Most mutations that cause PHS are located within the bHLH domain of the protein. For this reason, its interaction with DNA and other proteins is impaired^16–18^. Additionally, common *TCF4* gene variants are risk factors for non-psychotic disorders like Fuchs’corneal endothelial dystrophy (FECD)^19,20^ and primary sclerosing cholangitis (PSC)^21^.

In eukaryotes, one of the most important organelles is the cell nucleus. The cytoplasmic and nuclear compartments of the cell are separated by a double protein-lipid layer containing nuclear pore complexes that allow for passive and active transport of molecules in two directions. Precise regulation and proper localization is crucial for the ability of protein to act as an active transcription factor^22^. Molecules not exceeding 40 kDa can be transported in a passive way^23^. However, larger particles shuttle by the nuclear pore complex (NPC) in an active way due to their interaction with Karyopherins, which recognize specific motifs within the sequence of transported proteins^24^. Importins recognize the nuclear localization signal (NLS), typically rich in basic amino acid residues, and exportins recognize the nuclear export signal (NES), which is less conservative. One of the characteristics of a classical NES is the presence of multiple leucine or isoleucine residues^25,26^. Leptomycin B (LMB) as an inhibitor of exportin-1 dependent transport from the nucleus to the cytoplasm and is often used in experimental verification of NES activity^27–29^. Detection of the NES and NLS is not simple and is unambiguous. These signal activities are not only dependant on the occurrence of specific amino acid residues, but also on the secondary structure of the protein, its flexibility and surface exposure^26^, post-translational modifications, and interactions with other proteins^25^.

The first report concerning subcellular localization of TCF4 showed this transcription factor to be only in the nucleus^3^, while related proteins from the class I bHLH family, like E2a and HEB, were detected both in nuclear and cytoplasmic fractions of human embryonic stem cells^30^. Detailed studies examining the expression of isoforms of TCF4 that differ in their N-terminal and internal sequences revealed that isoforms vary in terms of their subcellular localization. The longest isoform B presented a strictly nuclear localization, while others were present in both the nuclei and cytoplasm of HEK 293 cells and human brain tissue^5^. Later on, Brandl *et al*.^31^ investigated TCF4 distribution in human colorectal carcinomas cells. They showed that TCF4 localization in the cytoplasm was positive in 76% of cases, while localization in the nucleus was positive in 51% of cases. Importantly, the cytoplasmic localization of TCF4 was correlated with better survival. Cytoplasmic localization of TCF4 was also observed by d’Rosario *et al*.^32^. In their research, TCF4 was only detected in the nuclei of some neurons, while in the cytoplasm (soma and dendrites) it was seen in most post-mitotic neurons. Sepp *et al*.^33^ did not agree with these results. They presented results of the overexpression of different isoforms of TCF4 in the primary neurons of rats, which were fully consistent with previously published results^5^. The authors also declared that the regulation of TCF4 by neuronal activity cannot be attributed to its signal-dependent nuclear import. Finally, in 2018, Jung *et al*.^34^ showed that TCF4 expression in the lateral (LA) and basolateral (BLA) nuclei in the amygdala of young adult mice was nuclear, while in the central amygdala nucleus (CEA) the TCF4 was located in fibers and not in the nucleus. The authors suggested the possibility that TCF4 activity could be modulated by nuclear-cytoplasmic translocation.

Due to the discrepancies presented above, we decided to systematically analyse the presence of putative NLSs and/or NESs and their role in subcellular trafficking of TCF4-B isoforms. We demonstrate that TCF4 contains an NLS that is partially overlapping with two LMB-sensitive NESs in the bHLH domain. The location of the NLS and NESs in close proximity raises the possibility of alternating the activity of these signals and suggests that the interaction of the NLS with importin prevents the interaction of NESs with exportin and vice versa. This dynamic might be important in the regulation of TCF4 cellular shuttling. Interestingly, our experiments showed no NES activity for the previously suggested predicted motif in the N-terminal part of protein (aa 10–19)^32^. Additionally, we proposed that the previously documented NLS (156–178 aa)^5^ might function as a Nucleolar localization signal (NoLS). *In silico* analysis shows high conservation of NLSs and NESs between TCF4 and related mammalian proteins, indicating the importance of these sequences for the general regulation of class I bHLH transcription factors subcellular shuttling, and in consequence, also their function.

## Results

### Subcellular localization of TCF4 in COS-7 and N2a cells

**Figure 1:**
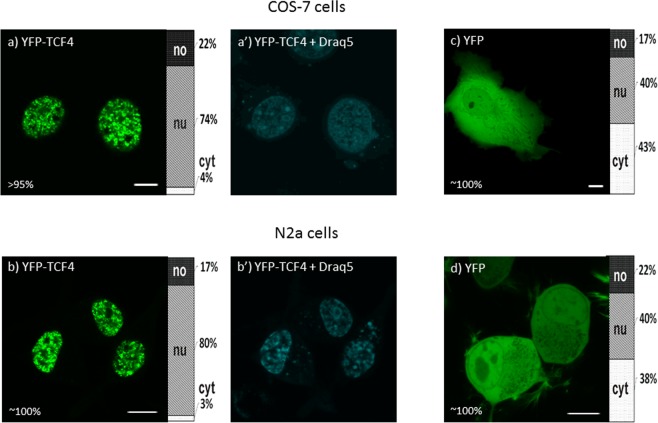
Subcellular distribution of full-length TCF4. Subcellular localization of YFP-tagged TCF4 was analysed by confocal microscopy 20-24 h after transfecting COS-7 and N2a cells. Draq5 was added to the cells for DNA visualization. Ratios between mean fluorescence intensity in cytoplasmic, nuclear and nucleolar compartments are presented as an accumulated bar graph (no- nucleolus; nu- nucleus, cyt- cytoplasm). (**a**) Representative images (single confocal plane) of the subcellular distribution of YFP-TCF4 (**a**-b’) and YFP (**c**-**d**) in COS-7 and N2a cells. Bar, 10 µm. no- nucleolus; nu- nucleus, cyt- cytoplasm.

Previously it was shown that green fluorescent protein (GFP) can be used for monitoring protein expression and localization in living organisms^35^, and yellow fluoresce protein (YFP) did not influence localization of the related TCF4 and E47 protein^36^. Twenty-four hours after transient transfection of the COS-7 and N2a cells, we analyzed the subcellular localization of N-terminally YFP-tagged full-length TCF4 using fluorescent microscopy. The expression of YFP-TCF4, and in further experiments YFP-tagged TCF4 mutants in the COS-7 cells, was confirmed by western blot analysis using an anti-GFP antibody (Fig. S1). As the expression of TCF4 was documented in various tissues^7^, we decided to use two different cell lines: COS-7 cells used previously for bHLH protein localization studies^37,38^ and Neuro2A mouse neuroblastoma (N2a) cells with a documented expression of TCF4^5^. The distribution of TCF4 fused with YFP in more than 95% of COS-7 cells was strictly nuclear (Fig. 1a,a’), however there were some cells (less than 5%) presenting fluorescence not only in the nucleus, but also in the cytoplasm in the puncta that were non-uniformly distributed in the cytoplasm of cells (Fig. S2). In the case of the N2a cells, the expression of YFP-TCF4 was exclusively nuclear (Fig. 1b,b’). As proof that YFP does not influence TCF4 localization, we expressed the YFP protein alone in the COS-7 (Fig. 1 c) and N2a cells (Fig. 1d), which, as expected, resulted in ubiquitous localization. Differences in localization of TCF4, depending on the cell type and literature data presented here, led us to the hypothesis about the putative presence of the composite system of NLSs and NESs in TCF4. This hypothesis is substantiated by a previous report indicating that related proteins, E2a and HEB, can be observed in the nucleus and cytoplasm of human embryonic stem cells (hESCs)^30^.

### The N-terminal fragment of the canonical TCF4-B isoform do not possess an active NES

**Figure 2:**
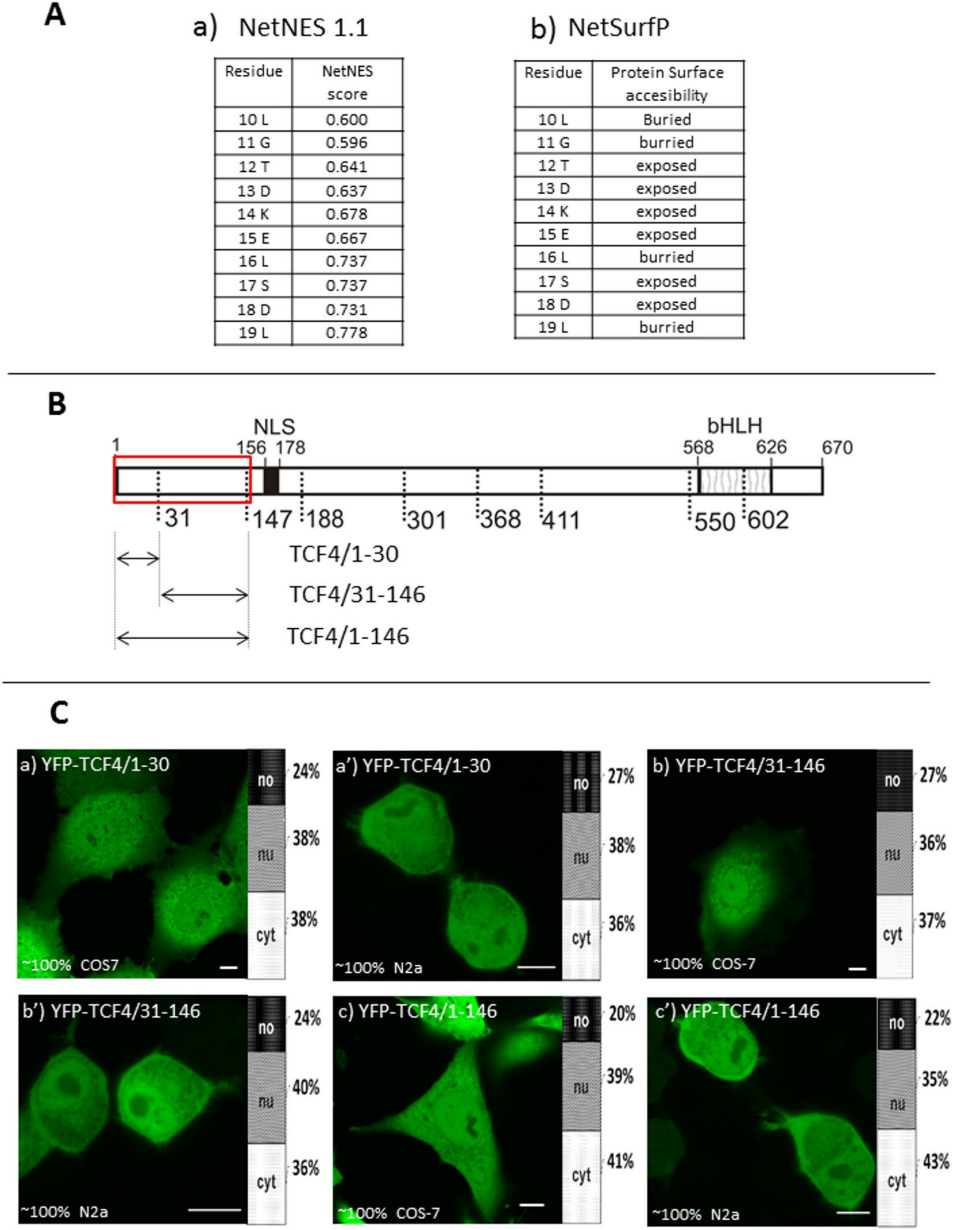
Subcellular distribution of deletion mutants (1-146aa) of TCF4 (**A**) Results of NetNES1.1(a) and NetSurfP (b) Predictors (**B**) Schematic representation of TCF4 protein. Regions of the TCF4 (NLS and SMART predicted bHLH) are depicted using different patterns and upper numbering of aa residues. The length of each domain in the diagram is arbitrary. Bottom aa residues refer to first aa residues of all TCF4 fragments used in this study. Region of actual studied area of TCF4 is shown by the red rectangle. Expressed deletion mutants of TCF4 are depicted as arrows and particular deletion mutants are depicted as arrows. The same fashion is used for Figs 2–4 and 6–10 (**C**) Subcellular distribution of deletion mutants of TCF4. Subcellular localizations of the expressed proteins were analysed by confocal microscopy 20-24 h after transfecting COS-7 and N2a cells. Representative images (single confocal plane for confocal microscopy) of subcellular distribution of the derivatives of the TCF4/1-146 area. Bar, 10 µm. Ratios between mean fluorescence intensity in cytoplasmic, nuclear and nucleolar compartments are presented as an accumulated bar graph (no- nucleolus; nu- nucleus, cyt- cytoplasm). (a,a’) YFP-TCF4/1-30, (b,b’) YFP-TCF4/31-146, (c,c’) YFP-TCF4/1-146, (**C**) Results of NetNES1.1(a) and NetSurfP (b) Predictors.

It was previously suggested that the N-terminal region of TCF4 could contain an active NES, however experimental verification of prediction was not performed^39^. We repeated *in silico* analysis using currently available NES predictors and obtained the same positive results for 10–19 aa (Fig. 2Aa). To experimentally test the activity of putative NES, we prepared deletion fragments comprising amino acid residues 1–30, 31–146, and 1–146 fused C-terminally to YFP (Fig. 2B). The expression of YFP-TCF4/1–30 (Fig. 2Ca,a’), YFP-TCF4/31–146 (Fig. 2Cb,b’) and YFP-TCF4/1–146 (Fig. 2Cc,c’) in the COS-7 and N2a cells resulted in the ubiquitous distribution of fluorescence throughout the whole cell. These results show that the sequence predicted by the NetNES server (Fig. 2Aa) is not an active NES in this region. The lack of activity can be explained by results of *in silico* analysis by NetSurfP, showing that leucine residues important for classical NES are predicted to be not exposed to the surface of the protein (Fig. 2Ab).

### Previously determined NLS can act as NoLS

**Figure 3:**
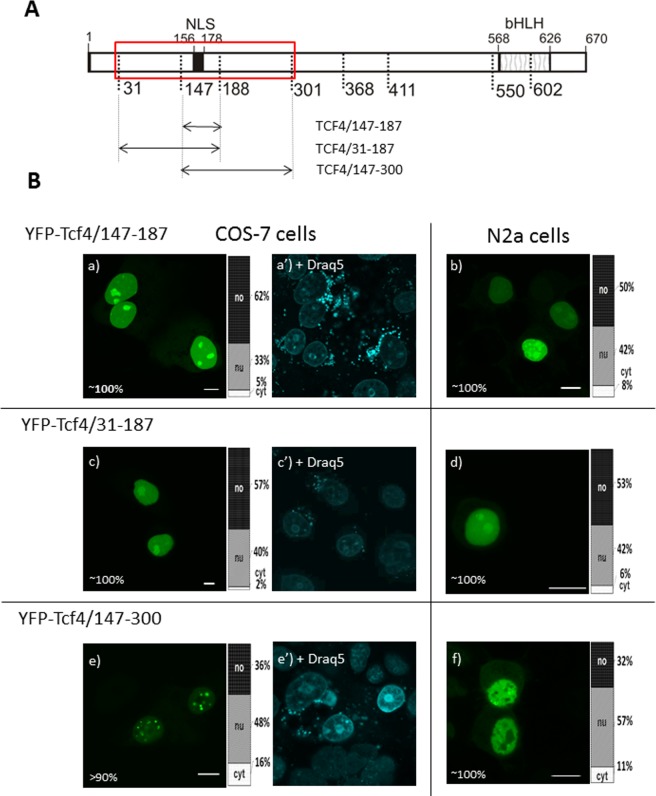
Subcellular distribution of deletion mutants (147-300aa) of TCF4 (**A**) Schematic representation of TCF4 protein. Region of the studied area of TCF4 is shown by the red rectangle. Expressed deletion mutants of TCF4 are depicted as arrows. The length of each domain in the diagram is arbitrary. (**B**) Subcellular distribution of deletion mutants of TCF4. Subcellular localizations of the expressed proteins were analysed by confocal microscopy 20-24 h after transfecting COS-7 and N2a cells. Representative images (single confocal plane) of subcellular distribution of the derivatives of the TCF4/31-300 area. Bar, 10 µm. Draq5 was added to the cells for DNA visualization. Ratios between mean fluorescence intensity in cytoplasmic, nuclear and nucleolar compartments are presented as an accumulated bar graph (no- nucleolus; nu- nucleus, cyt- cytoplasm). (a,a’,b) YFP-TCF4/147-187, (c,c’,d) YFP-TCF4/31-187, (e-f) YFP-TCF4/147-300.

When testing the localization of different isoforms of TCF4, Sepp with co-workers^5^ identified bipartite NLS in the region of 8–9 exons of TCF4 comprising amino acid residues 156–178 in the TCF4-B isoform. This NLS was shown to be conserved among E-proteins^5,40^. We prepared YFP-tagged truncation mutants of TCF4 containing this area (Fig. 3A). The expression of YFP-TCF4/147–187 resulted in a strictly nuclear fluorescence in the COS-7 cells (Fig. 3Ba). Importantly, nucleolar localization was observed, which was proven by the staining with Draq5 dye that was used for visualization of chromatin and nucleoli positions (Fig. 3Ba’). Moreover, in the case of YFP-TCF4/147–187 expression in the N2a cells, we observed fluorescence in both the nuclei and nucleoli (Fig. 3Bb). Interestingly, the expression of YFP-TCF4/31–187 resulted in the same results for both the COS-7 (Fig. 3Bc,c’) and N2a (Fig. 3Bd) cells, while YFP-TCF4/147–300 presented only nuclear, and not nucleolar localization. Instead, some punctate pattern appeared in the nuclei of the COS-7 cells (Fig. 3Be,e’), and to a lesser extent in the N2a cells (Fig. 3Bf).

Previous research by Słomnicki *et al*.^41^ detected the nucleolar presence of TCF4 in primary cultures of forebrain neurons and astrocytes. We performed *in silico* prediction of the nucleolar localization signal (NoLS) in TCF4 with a Nucleolar localization sequence Detector (NoD) and got negative results. However, prediction of the NoLS is characterized by low accuracy and needs experimental verification^42^. A typical characteristic of the NoLS is the high presence of basic amino acid residues that are located in the alpha-helical and easily accessible region on the surface of the protein. There are three known classes of NoLSs and NLSs: NoLS-only signals, NLS-only signals and the joint NoLS-NLS region containing an overlapping NLS and NoLS^42^. As all characteristics necessary for acting as a NoLS possess a NLS (156–178aa) in the TCF4, we propose that this NLS may present additional NoLS activity that is regulated by an unknown mechanism (potentially posttranslational modification or interaction with a masking NoLS activity partner) that maybe uses some of the amino acid residues present in the 188–300 area of the TCF4.

### No active localization signal exists in the central part of TCF4

**Figure 4:**
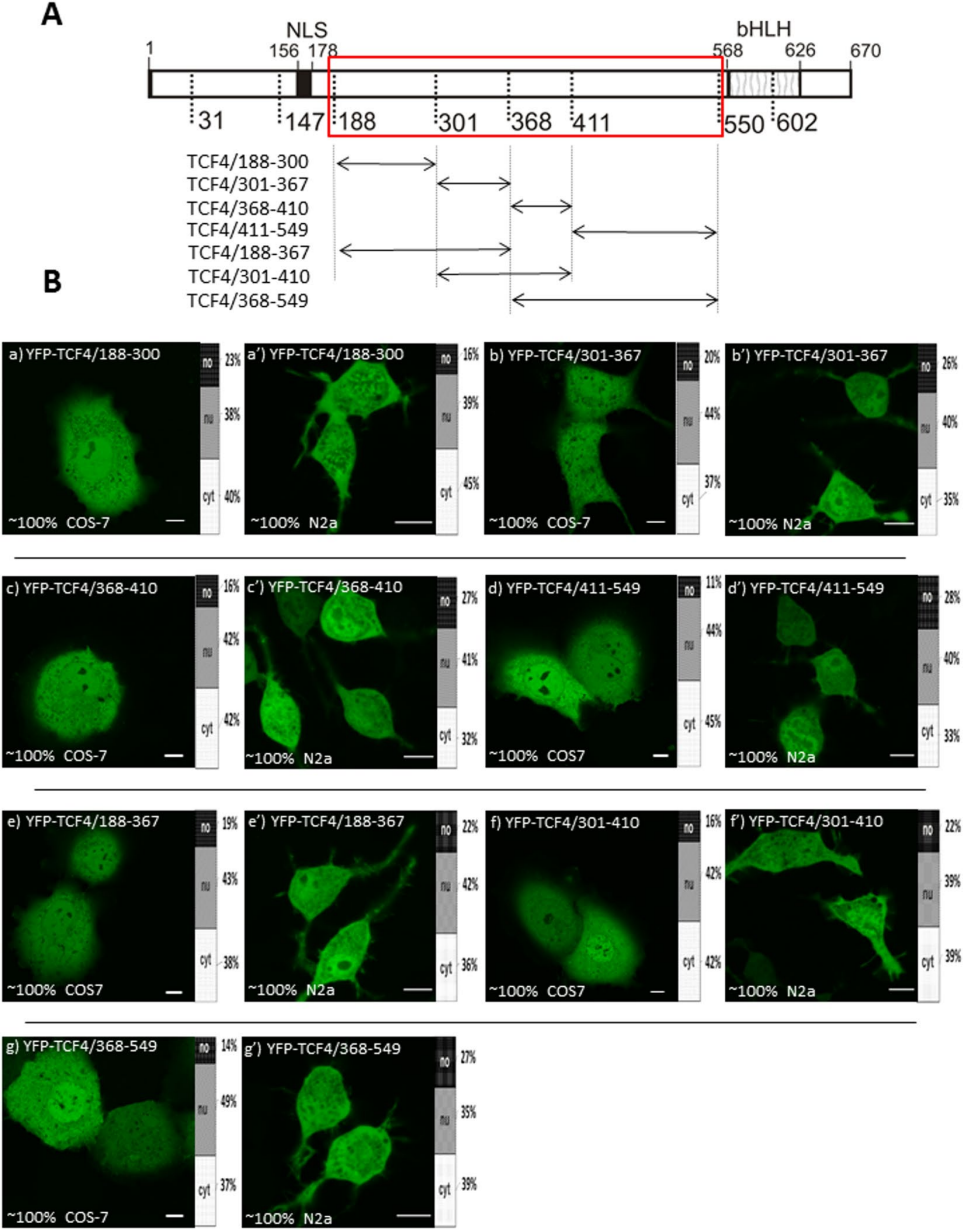
Subcellular distribution of deletion mutants (188-549aa) of TCF4 (**A**) Schematic representation of TCF4 protein. Region of the studied area of TCF4 is shown by the red rectangle. Expressed deletion mutants of TCF4 are depicted as arrows. The length of each domain in the diagram is arbitrary. (**B**) Subcellular distribution of deletion mutants of TCF4. Subcellular localizations of the expressed proteins were analysed by confocal microscopy 20-24 h after transfecting COS-7 and N2a cells. Representative images (single confocal plane for confocal microscopy) of subcellular distribution of the derivatives of TCF4/188-549 area. Bar, 10 µm. Ratios between mean fluorescence intensity in cytoplasmic, nuclear and nucleolar compartments are presented as an accumulated bar graph (no- nucleolus; nu- nucleus, cyt- cytoplasm). (a,a’) YFP-TCF4/188-300, (b,b’) YFP-TCF4/301-367, (c,c’) YFP-TCF4/368-410, (d,d’) YFP-TCF4/411-549, (e,e’) YFP-TCF4/188-367, (f,f’) YFP-TCF4/301-410, (g,g’) YFP-TCF4/368-549.

NLS predictors recognize classical signals rich in basic amino acid residues, while the last few years has also seen the discovery of proline-tyrosine-NLSs^43^. Moreover, NES appears to be a complex and diverse signal, not always being a classical leucine-rich motif^44^. To systematically test the presence of potential sequences that influence subcellular localization in the central region of TCF4, we analysed fragments of this region fused C-terminally to YFP. These TCF4 fragments were defined according to the secondary structure prediction, which was performed using PSIPRED (data not shown). Eventually, four fragments, encompassing residues: 188–300, 301–367, 368–410 and 411–549 were selected (Fig. 4A). The expressed proteins: YFP-TCF4/188–300 (Fig. 4Ba,a’), YFP-TCF4/301–367 (Fig. 4Bb,b’), YFP-TCF4/368-410 (Fig. 4Bc,c’) and YFP-TCF4/411-549 (Fig. 4Bd,d’) presented uniform distribution between the nucleus and cytoplasm in the COS-7 and N2a cells. To guarantee that we did not disrupt any structural motif in the constructs presented above, we also prepared fused versions of the previously selected fragments (Fig. 4A). Similarly to the results described above, YFP-tagged TCF4/188-367 (Fig. 4Be,e’), YFP-TCF4/301-410 (Fig. 4Bf,f’) and YFP-TCF4/368-549 (Fig. 4Bg,g’) were ubiquitously distributed in the COS-7 and N2a cells. As no NLS or NES motif was predicted in this area, this result proved the lack of active subcellular localization signals in this region under the used conditions.

### The bHLH domain of TCF4 possesses an overlapping NLS and two NES motifs

**Figure 5:**
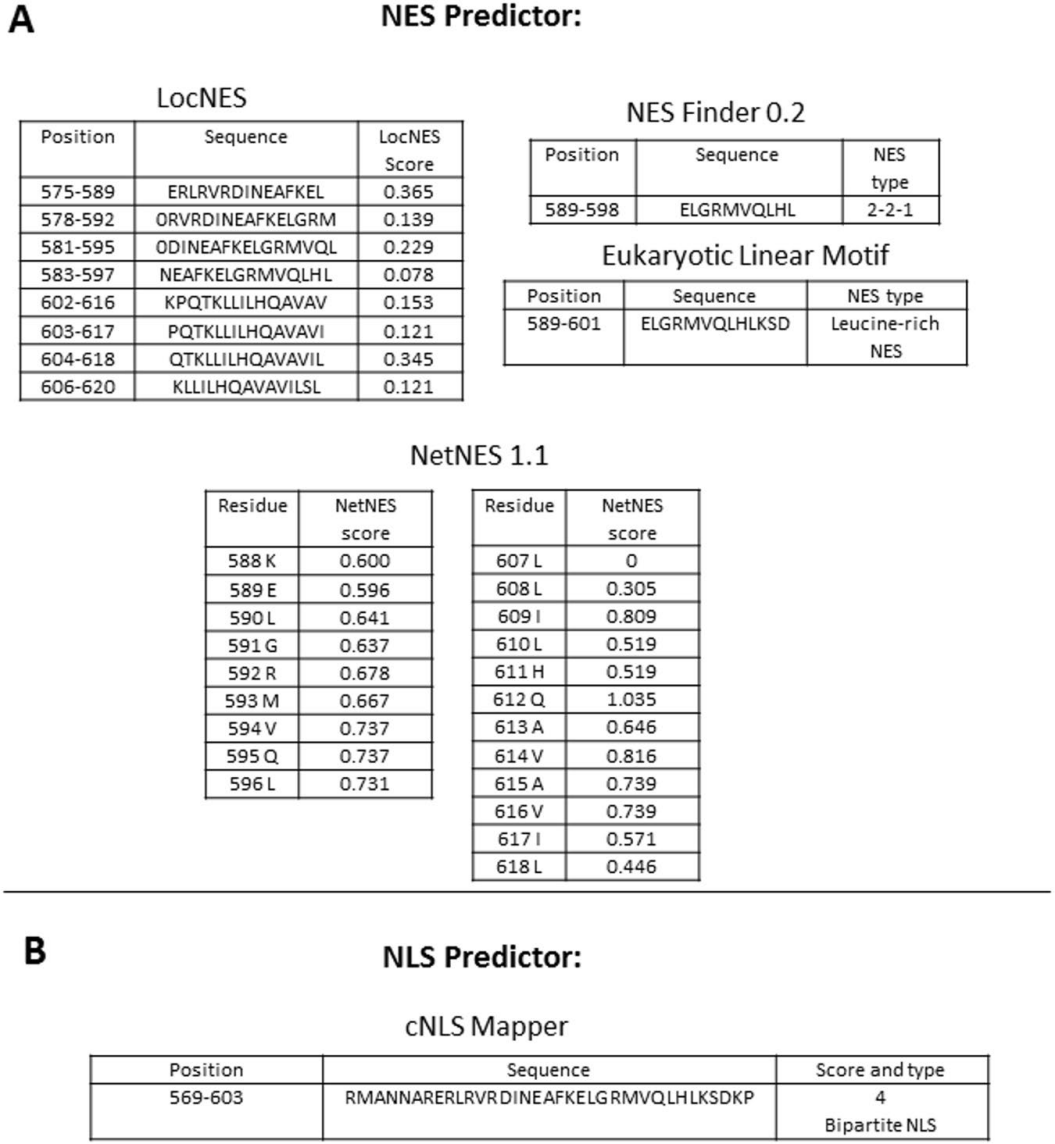
NES and NLS prediction in the bHLH domain of TCF4. (**A**) Results of NES prediction using LocNES, NES Finder 0,2, NetNES1.1. and ELM servers. (**B**) Results of NLS prediction using the cNLSMappes server.

It was previously shown that the full length GFP tagged bHLH domain of TCF4 was present in the nucleus and cytoplasm of the HEK293 cells, while the expression of the separated N- and C-terminal parts of this domain resulted in cytoplasmic localization of both truncations^5^. As previously published data did not unambiguously define the molecular features that affect subcellular distribution of the bHLH domain, we decided to perform a detailed study of this area. We asked the question about the presence of putative NESs and NLSs in fragments containing bHLH using different predictors. According to CDD (conserved domain database), a resource for the annotation of functional units in proteins, homology area of bHLH of TCF4 was shown for aa residues 553-634 in contrast to predicted by Smart (568-626) (see Fig. 5A) and Prosite (567-620) (Fig. S3). Due to this, we decided to add additional aa residues to both the N- and C-terminus of the predicted bHLH in order to be sure that we did not destroy any structural motif. In this paper we refer to the bHLH domain as area 550-670 aa, additionally divided for the N-terminal (550-601) and C-terminal (602-670) part (Fig. 4A).

Results suggested the presence of NESs in two fragments – one comprising the N-terminal part of the bHLH, and one comprising the C-terminal part of the bHLH (Fig. 5A). Additionally, we performed a prediction of the putative NLS with a cNLS Mapper and got a positive result for aa 569-603 (Fig. 5B) overlapping predicted NESs. We decided to experimentally test the activity of all the predicted signals.

**Figure 6:**
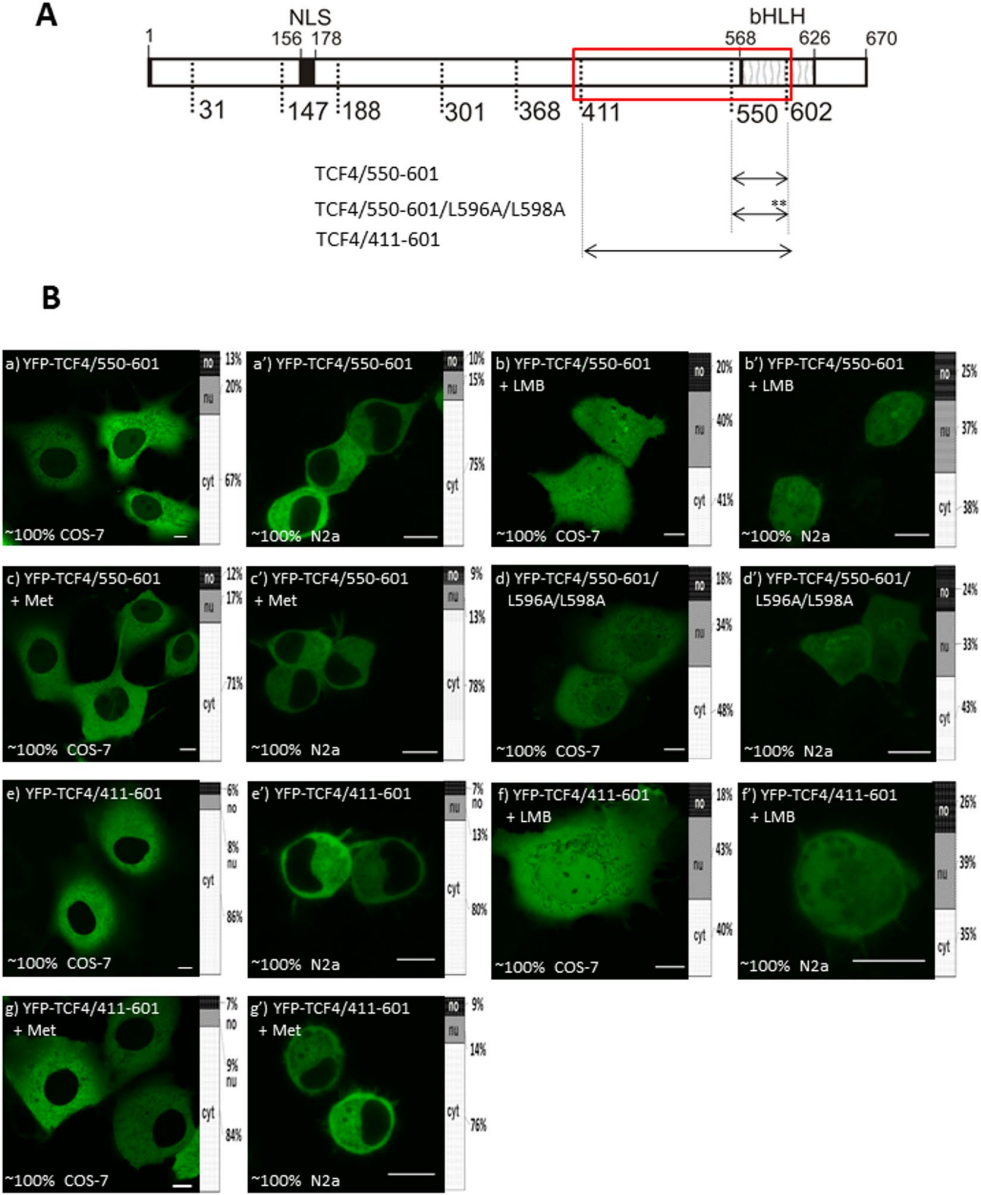
Subcellular distribution of deletion mutants (411-601aa) of TCF4 (**A**) Schematic representation of TCF4 protein. Region of the studied area of TCF4 is shown by the red rectangle. Expressed deletion mutants of TCF4 are depicted as arrows. Point mutations are depicted as stars. The length of each domain in the diagram is arbitrary. (**B**) Subcellular distribution of the deletion and point mutants of TCF4. Subcellular localizations of the expressed proteins were analysed by confocal microscopy 20-24 h after transfecting COS-7 and N2a cells in the absence or presence of additional factors such as LMB or methanol. Representative images (single confocal plane for confocal microscopy) of subcellular distribution of the derivatives of TCF4/411-601 area. Bar, 10 µm. Ratios between mean fluorescence intensity in cytoplasmic, nuclear and nucleolar compartments are presented as an accumulated bar graph (no- nucleolus; nu- nucleus, cyt- cytoplasm). (a,a’) YFP-TCF4/550-601, (b,b’) YFP-TCF4/550-601 after LMB addition, (c,c’) YFP-TCF4/550-601 after methanol addition, (d,d’) YFP-TCF4/550-601/L596A/L598A, (e,e’) YFP-TCF4/411-601, (f,f’) YFP-TCF4/411-601 after LMB addition, (g,g’) YFP-TCF4/411-601 after methanol addition.

First, we prepared the YFP-TCF4/550-601 construct (Fig. 6A) comprising the N-terminal fragment of the bHLH. As expected and according to the prediction of the NES in this area, localization of this fragment was dominantly localised in cytoplasm of both the COS-7 and N2a cells (Fig. 6Ba,a’). Leptomycin B (LMB) is known as an inhibitor of protein transport from the nucleus to the cytoplasm, which depends on a leucine-enriched sequence known as classical NES by interaction with exportin-1, which is responsible for protein export from the nucleus^27–29^. As predicted, the NESs were rich in leucine residues (see Fig. 5A). We used LMB to verify our hypothesis regarding the presence of an NES in the N-terminus of the bHLH. The presence of LMB resulted in a fluorescence signal that was present throughout the whole cell for both the COS-7 (Fig. 6Bb) and N2a (Fig. 6Bb’) cells. To exclude the influence of LMB solvent (70% methanol), we added methanol as a control and observed cytoplasmic localization of YFP-TCF/550-601 (Fig. 6Bc,c’) similarly to results without LMB addition. These results substantiated our hypothesis about the presence of active NES. By analysing different predictor results, we hypothesized that a sequence comprising aa 589-598 might be responsible for the NES activity in this fragment. To prove this hypothesis, we prepared a mutant where L596 and L598 were substituted by A (YFP-TCF4/550-601/L596 A/L598A; Fig. 6A). Localization of this mutant changed from being dominantly cytoplasmic for the unmodified fragment to being distributed in both the nucleus and cytoplasm of the cell. Interestingly, the COS-7 and N2a cells presented a slightly higher fluorescence signal in the cytoplasm (Fig. 6Bd,d’), suggesting some residual activity of NES. The N-terminal extension of NES containing the 550-601 fragment by neighbouring 411-549 aa (YFP-TCF4/411-601; Fig. 6A) presented cytoplasmic localization in both the used cell lines (Fig. 6Be,e’), demonstrating an active NES. The addition of LMB shifted localization to being equally distributed in the nucleus and cytoplasm in the COS-7 and the N2a cells (Fig. 6Bf,f’). Methanol as the control had no influence on localization pattern (Fig. 6Bg,g’).

**Figure 7:**
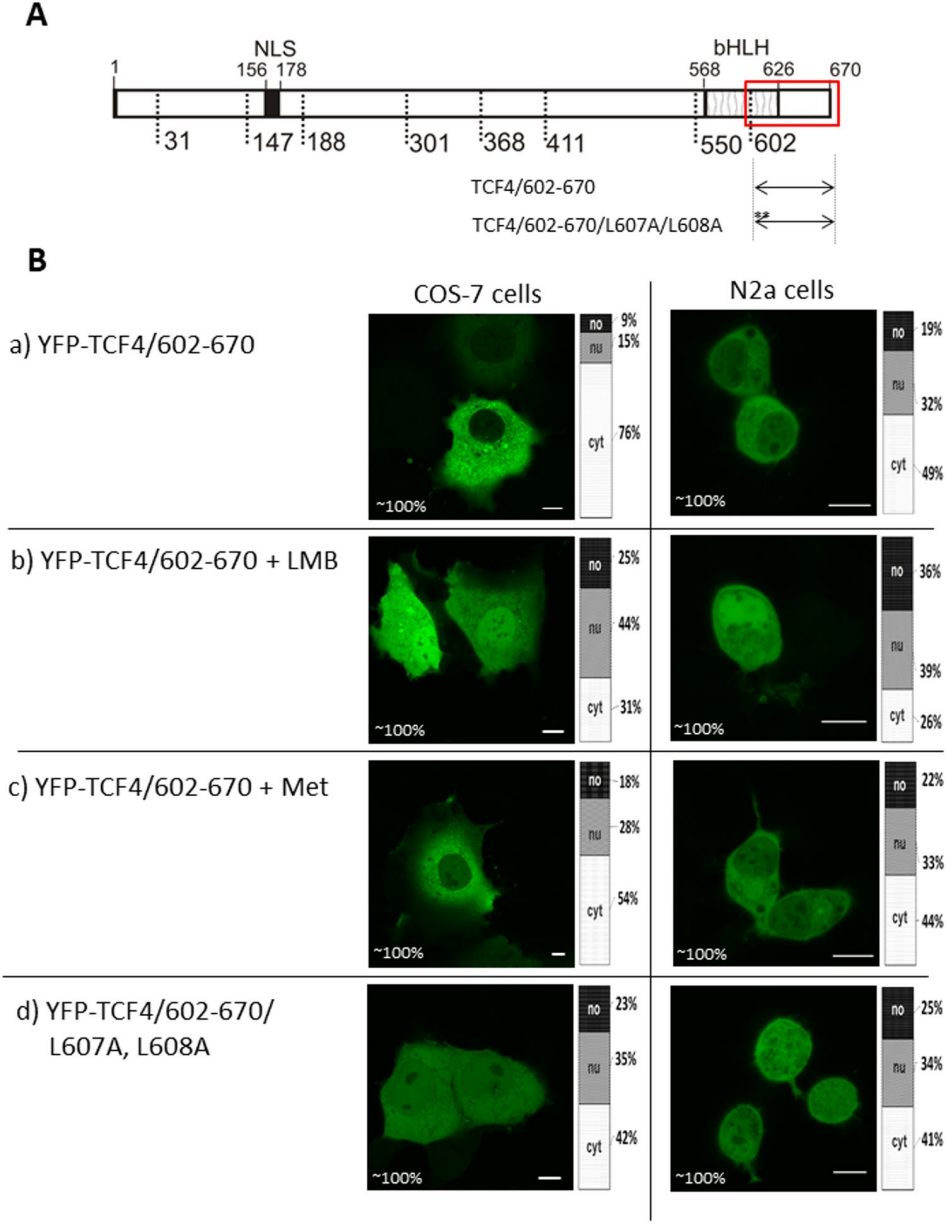
Subcellular distribution of the deletion mutant (602-670aa) of TCF4 (**A**) Schematic representation of TCF4 protein. Region of the studied area of TCF4 is shown by the red rectangle. Expressed deletion mutants of TCF4 are depicted as arrows. Point mutations are depicted as stars. The length of each domain in the diagram is arbitrary. (**B**) Subcellular distribution of deletion and point mutants of TCF4. Subcellular localizations of the expressed proteins were analysed by confocal microscopy 20-24 h after transfecting COS-7 and N2a cells in the absence or presence of additional factors such as LMB or methanol. Representative images (single confocal plane for confocal microscopy) of subcellular distribution of the derivatives of TCF4/602-670 area. Bar, 10 µm. Ratios between mean fluorescence intensity in cytoplasmic, nuclear and nucleolar compartments are presented as an accumulated bar graph (no- nucleolus; nu- nucleus, cyt- cytoplasm). (a) YFP-TCF4/602-670, (b) YFP-TCF4/602-670/L607A/L608A (c) YFP-TCF4/602-670 after methanol addition, (d) YFP-TCF4/602-670 after LMB addition.

To verify the activity of the predicted NES in the C-terminal part of the bHLH domain, we prepared a YFP-TCF4/602-670 construct (Fig. 7A). The expression of this construct resulted in fully cytoplasmic fluorescence in the COS-7 cells and cytoplasm prevailing in the N2a cells (Fig. 7Ba). Next, we added LMB and observed a shift of localization from the cytoplasm to being equal in the cytoplasm and nucleus (Fig. 7Bb), presenting the inhibitory effect of LMB according to the prediction of the NES rich in L residues. Again, addition of methanol had no effect (Fig. 7Bc). After detailed analysis of the predictor results (see Fig. 5A), we chose the sequence comprising aa 604-618 as a hypothetic NES. To investigate this assumption, we prepared a mutant where L607 and L608 were substituted by A (YFP-TCF4/602-670/L607 A/L608 A; Fig. 7A). Localization of this mutant changed from being exclusively cytoplasmic, observed for the unmodified fragment, to being distributed equally in both compartments of the cell for bothCOS-7 and N2a cells (Fig. 7Bd).

Our results substantiate the thesis about the presence of one active NES (NES-1) in the N-terminal part of the bHLH (589-E**L**GRMVQ**L**H**L**-598; bold are leucine residues, bold and underlined are leucine substituted residue), and the presence of the second active NES (NES-2) in the C-terminal part of this domain (604-QTK**LL**I**L**HQAVAVI**L-**618).

**Figure 8:**
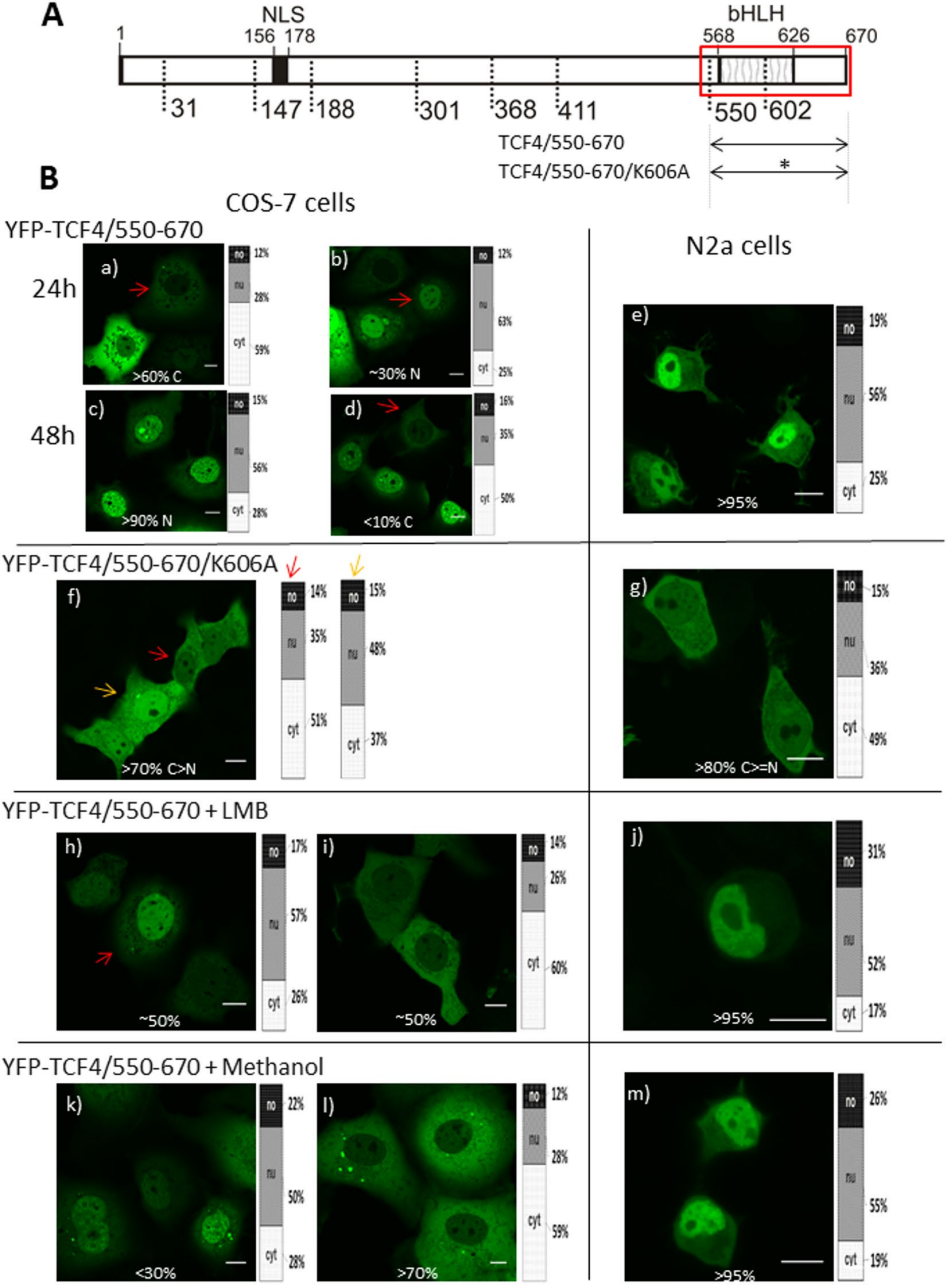
Subcellular distribution of the bHLH domain of TCF4 (**A**) Schematic representation of TCF4 protein. Region of the studied bHLH domain of TCF4 is shown by the red rectangle. Expressed deletion mutants of TCF4 are depicted as arrows. Point mutation is depicted as a star. The length of each domain in the diagram is arbitrary. (**B**) Subcellular localizations of the expressed proteins were analysed by confocal microscopy in COS-7 and N2a cells in the absence or presence of additional factors such as LMB or methanol. Representative images (single confocal plane for confocal microscopy) of subcellular distribution of the derivatives of TCF4/550-670 area. Bar, 10 µm. (a,b) YFP-TCF4/602-670 -24 h after transfection of COS-7 cells, (c, d) YFP-TCF4/602-670 - 48 h after transfection of COS-7 cells, (e) YFP-TCF4/602-670 - 24 h after transfection of N2a cells, (f) YFP-TCF4/550-670/K606A 24 h after transfection of COS-7 cells, (g) YFP-TCF4/550-670/K606A 24 h after transfection of N2a cells, (h, i) YFP-TCF4/602-670 24 h after transfection of COS-7 cells in the presence of LMB, (j) YFP-TCF4/602-670 24 h after transfection of N2a cells in the presence of LMB, (k, l) YFP-TCF4/602-670 24 h after transfection of COS-7 cells in the presence of methanol, (m) YFP-TCF4/602-670 24 h after transfection of N2a cells in the presence of methanol,

Surprisingly, the expression of YFP-TCF4/550-670 comprising bHLH (Fig. 8A) resulted in inconsistent results, as the COS-7 cells presented highly variable pattern of localization from only cytoplasmic, by distributed between cytoplasm and nucleus, to mainly nuclear. Repeated experiments have revealed the dependence of localization from the time after transient transfection. Microscopy imaging 24 h after transfection of the COS-7 cells resulted in predominantly cytoplasmic localization (>60% of cells; Fig. 8Ba). However, for some cells we also observed equal distribution in both compartments of the cell (<10% cells; Fig. S2), and dominantly nuclear localisation for others (approximately 30% cells) (Fig. 8Bb). Repeated imaging 48 h after transfection showed a highly dominant nuclear localisation of the bHLH in more than 90% of cells (Fig. 8Bc), while in less than 10% of cells we could still observe fluorescence in the cytoplasm (Fig. 8Bd). In the case of the N2a cells, 24 h after transfection, dominant nuclear localisation was presented by more than 95% of cells (Fig. 8Be), and we observed cytoplasmic fluorescence in only some cells (<5%) (Fig. S2). All the above results suggest the presence of an active NLS in the bHLH-containing fragment. The cNLS Mapper predicted the region aa 569-603 as a putative bipartite NLS motif rich in basic amino acid residues (see Fig. 5B). Knowing that the N-terminal part of the bHLH (aa 550-601) presented strictly cytoplasmic localization (see Fig. 6Ba,a’), we deduced that basic amino acid residues K602 (predicted as part of NLS) and K606 (not predicted as part of the NLS, however situated in close proximity to the predicted NLS) in the C-terminal part of the bHLH are indispensable for this NLS activity. For this reason we decided to substitute K606 for A (Fig. 8A). The expression of this point mutant (YFP-TCF4/55-670/K606A) in the COS-7 cells mainly resulted (>70%) in equal or slightly more intensive fluorescence in the cytoplasm (Fig. 8Bf). However, for some cells (<30%) we could observe localization at the same time in the cytoplasm, but could only observe this in some subnuclear punctate aggregates (Fig. S2). In the case of the N2a cells, localization was slightly more cytoplasmic (>80%) (Fig. 8Bg). For less than 20% of the N2a cells, the fluorescence signal was distributed equally through the cell (Fig. S2). Change of localization after substitution of K606 by A proved the importance of this amino acid residue for the detected NLS activity and substantiates the thesis that the motif 569-**R**MANNA**R**E**R**L**R**V**R**DINEAFKELG**R**MVQLHL**K**SD**K**PQT**K**-606 (bold are basic residues, bold and underlined is lysine substituted residue) is the second active NLS (NLS-2) in TCF4. Out results are consistent with the results of Lingbeck *et al*.^40^, who documented the presence of the second NLS in the basic region of other proteins from the I class bHLH family: E12 and E47.

Because of the non-uniform localization of the bHLH, probably caused by the overlapping NLS and NESs, we were interested in observing the influence of the NES activity inhibitor (LMB) on the localization of the expressed bHLH domain. YFP-TCF4/550-670 (comprising the bHLH and some additional aa) expressed with LMB in the COS-7 cells showed dual behaviour, each presented by about 50% of cells - domination in the nucleus (Fig. 8Bh) and cytoplasm (Fig. 8Bi). This presented a pattern similar to the expression without LMB, with a delicate shift in the direction of nuclear localization (from about 30% to 50% of the analysed cells). We are not able to clearly explain why the NESs in the bHLH of TCF4 are LMB sensitive when separated, while the whole bHLH domain is less sensitive. However, we hypothesise that complex multi-element regulation might be the reason for the differentiated response to LMB in the case of the whole bHLH domain (containing two NESs and an NLS) in comparison to the single separated element (NES). LMB addition to the N2a cells expressing YFP-TCF4/550-670 had no visible effect (Fig. 8Bj), while without LMB this fragment was already predominantly nuclear in more than 95% cells, as discussed above. Controlled addition of methanol as a solvent of LMB had no effect for both the COS-7 (Fig. 8Bk,l) and N2a (Fig. 8Bm) cells.

**Figure 9:**
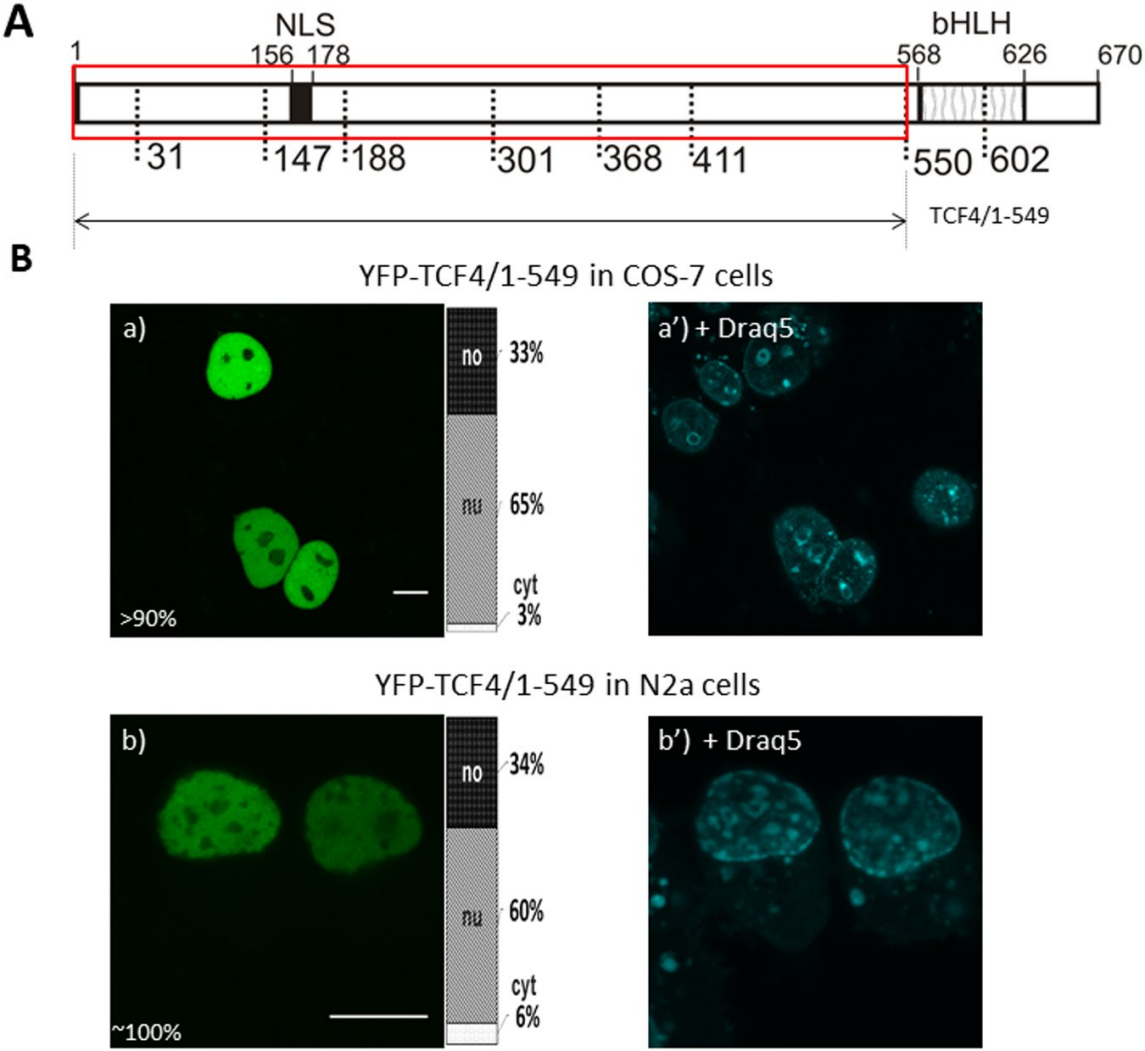
Subcellular distribution of TCF4 with deletion of the bHLH domain (**A**) Schematic representation of TCF4 protein. Region of the studied area of TCF4 is shown by the red rectangle. Expressed deletion mutant of TCF4 is depicted as an arrow. The length of each domain in the diagram is arbitrary. (**B**) Subcellular localizations of the expressed protein was analysed by confocal microscopy 20-24 h after transfecting COS-7 and N2a cells. Draq5 was added to the cells for DNA visualization. Representative images (single confocal plane) of subcellular distribution of the TCF4/1-549 area. Bar, 10 µm. Ratios between mean fluorescence intensity in cytoplasmic, nuclear and nucleolar compartments are presented as an accumulated bar graph (no- nucleolus; nu- nucleus, cyt- cytoplasm). (a,a’) YFP-TCF4/188-300 expressed in COS-7 cells, (b,b’) YFP-TCF4/188-300 expressed in N2a cells.

**Figure 10:**
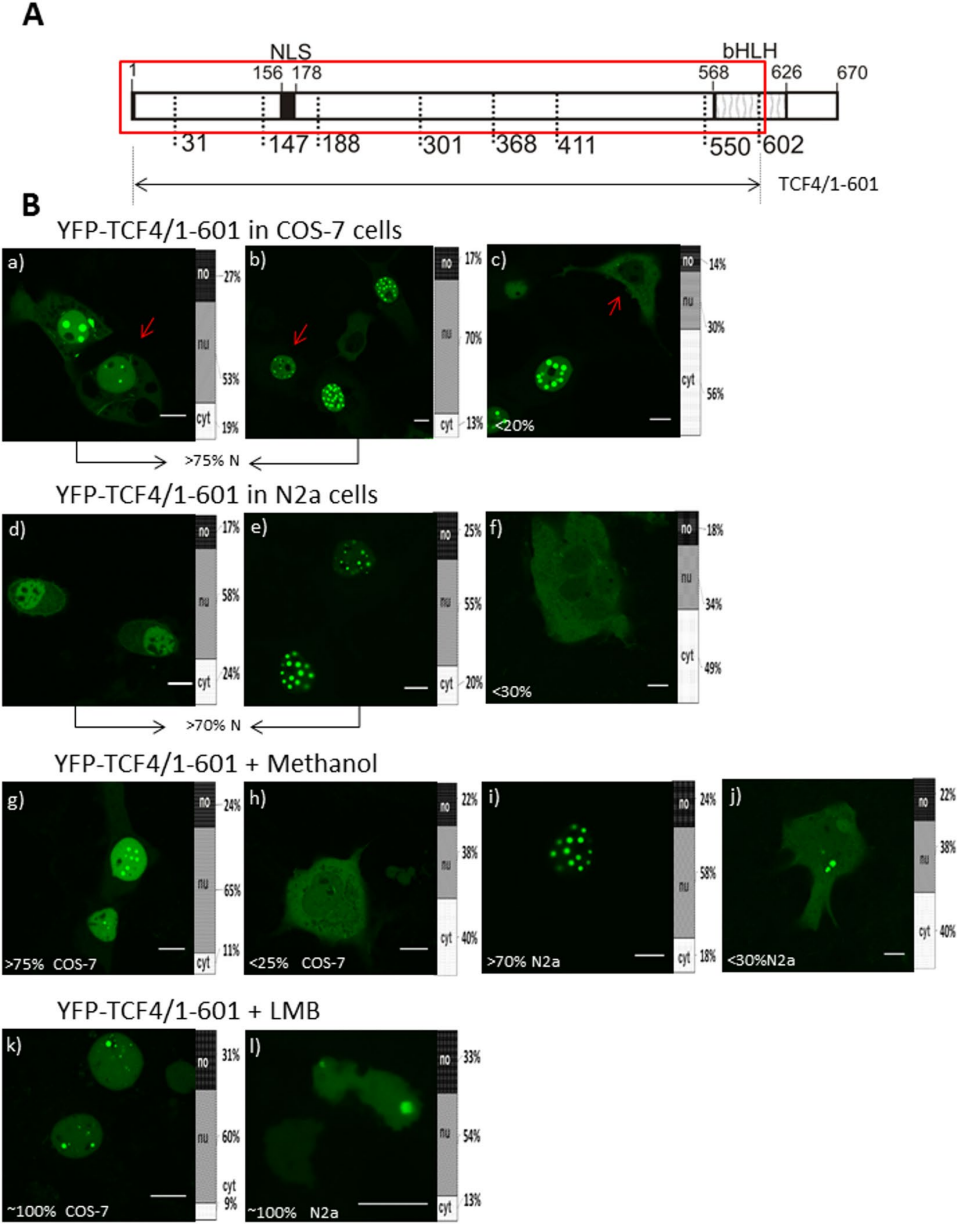
Hierarchy of NLS and NES signals in TCF4 (**A**) Schematic representation of TCF4 protein. Region of studied area of TCF4 is shown by the red rectangle. Expressed deletion mutant of TCF4 is depicted as an arrow. The length of each domain in the diagram is arbitrary. (**B**) Subcellular localizations of the expressed protein were analysed by confocal microscopy 20-24 h after transfecting COS-7 and N2a cells in the absence or presence of additional factors such as LMB or methanol. Representative images (single confocal plane for confocal microscopy) of subcellular distribution of the TCF4/1-601 area. Bar, 10 µm. Ratios between mean fluorescence intensity in cytoplasmic, nuclear and nucleolar compartments are presented as an accumulated bar graph (no- nucleolus; nu- nucleus, cyt- cytoplasm). (a-c) YFP-TCF4/1-601 in COS-7 cells, (d-f) YFP-TCF4/1-601in N2a cells (g,h) YFP-TCF4/1-601 after methanol addition in COS-7 cells, (i,j) YFP-TCF4/1-601 after methanol addition in N2a cells (k) YFP-TCF4/1-601 after LMB addition in COS-7 cells (l) YFP-TCF4/1-601 after LMB addition in N2a cells.

We intended to further investigate how particular signals in the bHLH domain impact the localization of TCF4. First, we prepared a C-terminally truncated mutant without the bHLH domain, instead containing only NLS-1 (YFP-TCF4/1-549; Fig. 9A). The expression of this YFP-tagged mutant in the COS-7 cells was strictly nuclear, as expected, and presented no fluorescence in the nucleoli (Fig. 9Ba,a’). Some of the COS-7 cells (<10%) also presented highly fluorescent puncta in the nucleus (Fig. S2). The YFP-TCF4/1-549 expression in the neuronal N2a cells resulted in a pattern analogical to the COS-7 cells (Fig. 9Bb,b’). Next, we created YFP-TCF4/1-601 (Fig. 10A) containing both NLS-1 and NES-1. The expressed protein distribution was extremely diversified in both the used cell lines (Fig. S4). The COS-7 cells were classified as cells presenting a predominantly nuclear (>75% of cells) and predominantly cytoplasmic (<20%) localization (Fig. 10Ba–c), and also presenting an equal distribution in both compartments (<5%) (Fig. S4). Additionally, in most cases of nuclear localization, some puncta with highly intensive fluorescence in the nucleus were also visible. Similar results were observed for the N2a cells, with >70% of the population presenting a nuclear (Fig. 10Bd,e) and <30% cytoplasmic signal of fluorescence (Fig. 10Bf). The addition of methanol did not change distribution (Fig. 10Bg–j), while LMB shifted the localization to be exclusively nuclear (Fig. 10Bk,l), proving the active role of NES-1 in directing protein to the final destination. We hypothesise that the intensive shift from the cytoplasm to the nucleus in the case of TCF4/1-601 in comparison to the TCF4/550-670 fragment comes from the presence of an NLS in the N-terminal part of the protein. Previously, it was shown that cells expressing TCF4 with a disrupted N-terminal NLS (158-172aa) presented a slightly dominant nuclear localization. This suggests the synergistic effect of NLSs from the N-terminal and C-terminal parts of the TCF4.

## Discussion

**Figure 11:**
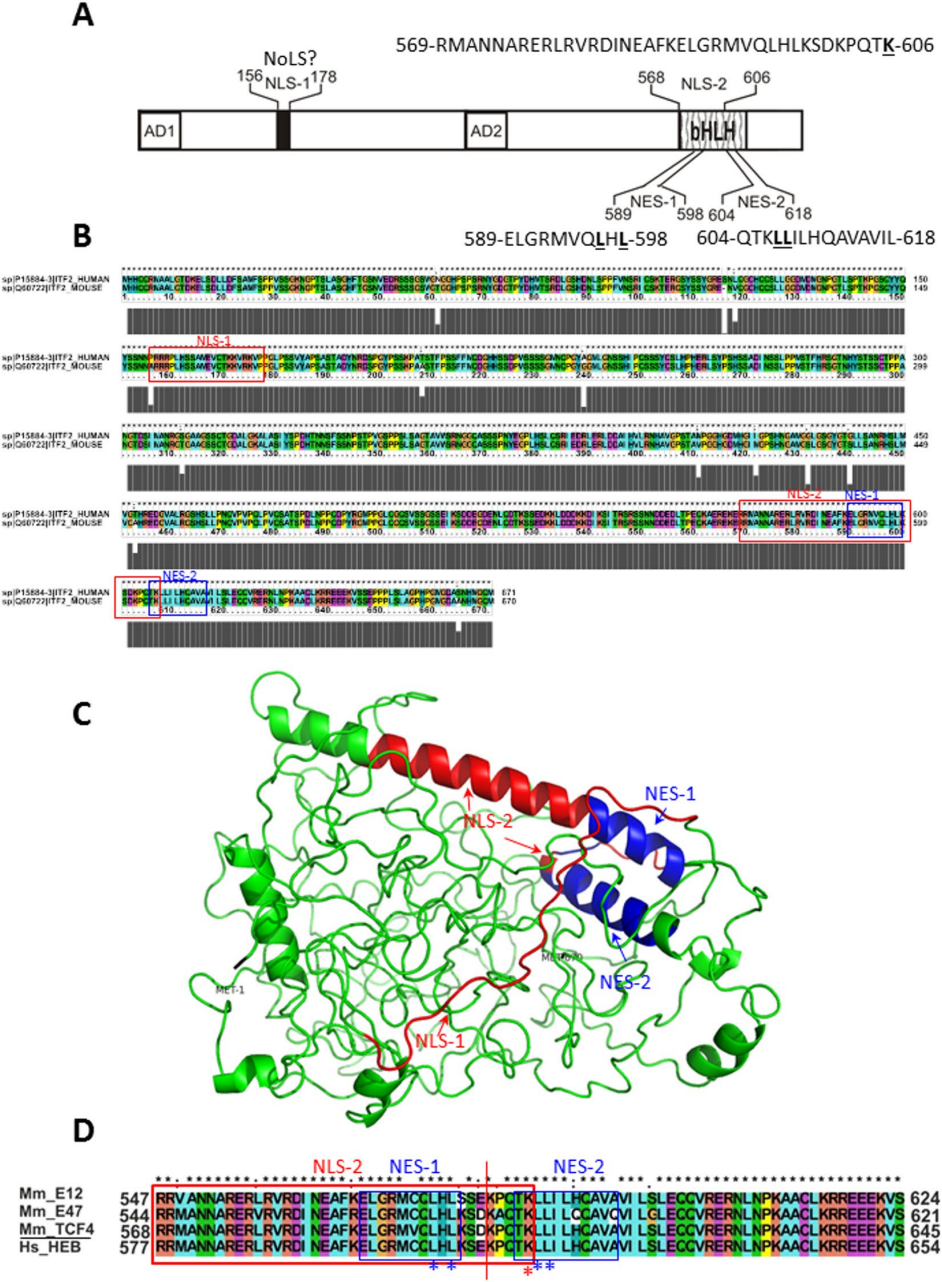
(**A**) Summary of NLSs and NESs in TCF4. Schematic representation of NLSs and NESs residing within the TCF4 protein structure. (**B**) Result of protein sequences alignment of TCF4-B+ isoforms of mouse and human TCF4 with ClustalX showing the conservation of TCF4 between these organisms. (**C**) Model of TCF4 generated by Phyre2. 113 residues (17% of protein) encompassing mainly the bHLH domain (aa 567-620) of TCF4 were modelled with a confidence higher than 90% using the templates: c2mh0A, c5c3lA, c2ql2A and c2ypbB. Remaining 601 residues (89%) of TCF4 lacking any template and predicted as disordered were modelled by ab initio. NLS-1 and NLS-2 are shown in blue. NES-1 and NES-2 are shown in red. (**D**) ClustalX sequence alignment of TCF4/568-645 region encompassing NLS-2, NES-1 and NES-2 in the bHLH domain with corresponding sequences of E12 (aa 547-624), E47 (aa 544-621) and HEB (aa577-654).

The subcellular distribution of TCF4 was proposed as one of the mechanisms regulating the function of this protein [63]. We showed that the previously documented NLS (NLS-1, Fig. 11A)^5^, located in the N-terminal part of the TCF4 protein (156-178aa), also possesses the putative activity of NoLS. We also showed that additional sequences may inhibit nucleolar localization. Our results are substantiated by a previous report stating that in cultured cortical- and hippocampus neurons and astrocytes, TCF4 in addition to nucleoplasm and pericarya was also present in the nucleoli. The authors also stated that PTHS–associated dominant-negative mutants R582P and R580Trp moderately reduced ribosomal biogenesis and general protein synthesis, pointing out the role of TCF4 in these processes^41^.

Additionally, we identified the activity of two NESs (NES-1 and NES-2) and an overlapping NLS (NLS-2) in the bHLH domain of TCF4 (Fig. 11A). We performed alignment of TCF4-B+ isoforms of mouse and human TCF4, which present a high sequence identity (Fig. 11B), pointing out the conservation of the mosaic pattern of overlaying localization signals with opposing activities between these two organisms. The presence of the NLS and NES in close proximity, or overlapping in the bHLH domain, was reported previously for the bHLH-PAS transcription factor aryl hydrocarbon receptor (AhR)^45^ and NPAS4^46^, while two NESs were shown in the bHLH of Sima (*Drosophila* homolog of HIF-1α)^47^. This suggests a more general function of the bHLH domain in the regulation of bHLH proteins shuttling. Laccase and Lefebvre proposed that overlaying of DNA binding motifs with localization signals allows for co-ordinate regulation^48^. We hypothesise that a system that regulates nucleocytoplasmic shuttling of these TF families is very complex and relies on many mutually dependent factors, both exportin-1 dependent and independent, like signal masking/unmasking, posttranslational modification and interaction with partner proteins such as calmodulin. This enables both precise and flexible signal transduction. Such complex multi-element regulation might also be the reason for the differentiated response to the LMB presence in the COS-7 cells during our experiments: high sensitivity in the case of separated NES-1 and NES-2 in the bHLH of TCF4, and low sensitivity in the case of the whole bHLH domain.

Importantly, it has previously been shown that point mutations of basic amino acid residues in the bHLH domain of TCF4 were often associated with the Pitt-Hopkins Syndrome. The first identified PTHS-associated heterozygous point mutations were R576W and R576Q^16,17^. Finally, PTHS-association was reported for: G358V, D535G, R569W, R572G, R574P, R574H, R576G, R576W, R576Q, R578P, R578H, R580W, R580Q, R582P, R587P, A610V and A614V TCF4 human mutants^49–53^. All the detected basic residue point mutants are localized in the NLS-2, while A610V and A614V are seen in the NES-2 sequence, thus suggesting the importance of localization signals in PTHS.

Localization studies of PTHS-connected point mutants performed previously, did not give consistent results, which was probably due to the different conditions of the performed experiments. Sepp *et al*.^52^ expressed wt and R576Q, R578H, R580W, R582P and A614 PTHS-associated mutants of TCF4-B^−^ proteins (isoform lacking RSRS) in HEK 293 cells, getting an analogical nuclear localization for both the wild type (wt) and all of the mutants. The expression of the bHLH tagged with GFP resulted in ubiquitous localization for wt and all the tested mutants. Forrest *et al*.^38^ performed the expression of G358V, D535G, R578P, R580W and A614V mutants of the TCF4-B^+^ isoform tagged with GFP in the COS-7 cells. Interestingly, while fluorescence for all the expressed proteins was visible only in the nucleus, the mutants R578P, R580W, and to a lesser extent A614V formed a small, spherical punctate, whose localization differed markedly from the distribution of the wild-type TCF4, G538V and D535G mutants. Similar results were obtained in SH- SY5Y neuroblastoma cells. We hypothesize that point mutations in the bHLH domain of TCF4, known for PTHS patients, influence pattern of distribution in nucleus and function of TCF4 probably by changes in DNA binding specificity. However, these single substitutions are not able to inhibit NLS-2 activity. In contrast, our experiments have led to the identification of amino acid residue K606 being indispensable for this NLS activity. Interestingly, this residue has not to date been connected to the PTHS disorder. Importantly, human aa residues located in the DNA binding region: R565, R576, E576, R578, R580, R587, K603 and K607 refer to murine aa residues R564, R575, E576, R577, R579, R586, K602 and K606 (Fig. 11B), which were pointed out by InterPro as being responsible for DNA binding

Localization signals can be structurally exposed, accessible to transport machinery or masked by interacting partners. To visualise the arrangement of the detected NLSs and NESs in the TCF4, we generated a 3D model of the TCF4 using Phyre2 (Fig. 11C). We also performed *in silico* analysis of TCF4 structure disorder, and 89% of the TCF4 sequence was predicted to be intrinsically disordered. The only area with a tendency to possess a more rigid structure was the bHLH domain. Interestingly, for this area we obtained a prediction of the highest surface accessibility (Fig. S3). Results of repeated in silico analyses for the selected C-terminal part of the protein (550-670aa) containing the bHLH domain showed that short intrinsic disorder regions could exist in the external parts of this domain (Fig. S5). The preferential location of NLSs and NESs in the intrinsically disordered regions (IDRs) of proteins was found to enable flexible and easily accessible interactions with their binding partners^54^.

IDRs are known to be the targets of intensive posttranslational modifications. Modifications such as phosphorylation, especially close to the NLS or NES, have been shown to regulate the intracellular distribution of proteins by activating or deactivating localization motifs^55^. Interestingly, phosphorylation of S448, located very close to the bHLH domain, by protein kinase A (PKA) was documented by Sepp and co-workers as being necessary for TCF4 transcriptional activity in cultured neurons and in the developing brain *in vivo*^33^. We performed predictions of TCF4 phosphorylation sites and found many putative sites of phosphorylation, especially in the N-terminal part of protein with probability higher then 0.5, located in NLS-1 area (aa 156-178): S145, S152, S162, T169, T182. We observed much far less predicted sites located in the C-terminus of protein. However, some of the S and T residues of TCF4, predicted as putative phosphorylation sites with a probability higher or equal to 0.8, are located in the bHLH domain (S600) or in close proximity to this domain (S546, S548, S549, T557, S645, S646, S652). The location of putative phosphorylation sites in the C-terminal part of protein is presented in Fig. S5.

Importantly, using three programs, the motif 542-SITRSR**S**SNND-552 was predicted with a high probability as the phosphorylated (S548) binding site for 14-3-3 proteins, which were shown to influence the localization of partner proteins with intrinsically disordered regions^56^. The ubiquitous family of 14-3-3 proteins is involved in the regulation of signal transduction^57^. Binding by 14-3-3 modulates the enzyme activity, subcellular localization, structure, stability and molecular interactions of partner proteins^58^. In this aspect, very interesting is the observation by *Liu et al*.^59^ that TCF4-B^−^ isoform, which encodes full-length protein without motif 545-RSRS-548, significantly reduced the promoter containing E-box activity, while the splice variant TCF4-B^+^ with RSRS did not affect this promoter activity in U1240MG cells. Taking all this into account, we hypothesize the importance of 14-3-3 interaction for the regulation of TCF4 localization, and in consequence, also this protein activity. On the other hand, we cannot exclude that a lack of phosphorylated S548 is the reason for the unbalanced and highly differentiated subcellular localization of the expressed TCF4/550-670.

The other mechanism for controlling TCF4 activity by differential subcellular localization could be Ca^2+^-dependent interaction with calmodulin, which was proposed as an important factor in the regulation of various target genes and cellular functions^60,61^. Though direct experimental data of how Ca^2+^ could modulate TCF4 activity in neurons are still lacking, there are some hypothesis based on studies with class I and II bHLH factors about Ca^2+^-regulated shuttling of TCF4 from the cytoplasm to the nucleus^62^. Interestingly, it was shown that calmodulin binds to the basic sequence of E12, E47 and TCF4^63^, raising the possibility that calmodulin binding interferes with the activity of localization signals existing in this area.

In this paper, we documented the presence of multiple localization signals with opposing activities, which suggests the complex and precisely balanced regulation of TCF4 subcellular shuttling by masking and unmasking of different localization signals in the bHLH domain by interacting partners and posttranslational modifications. We performed alignment of the TCF4/568-645 region, encompassing NLS-2, NES-1 and NES-2 in the bHLH domain with corresponding sequences of E12 (aa 547-624), E47 (aa 544-621) and HEB (aa577-654). Sequence homology in these areas is very high (Fig. 11D), and we therefore propose that not only NLS-2, confirmed by Lingbeck *et al*. [69], but also NES-1 and NES-2 activities might be present in all mammalian E proteins. We therefore believe that these signals are important elements that regulate dynamic exchange between the subcellular compartments of not only TCF4, but generally bHLH class I transcription factors. The complex interplay of regulatory mechanisms of the intracellular localization of TCF4 and other I class bHLH transcription factors requires further studies *in vivo*.

## Materials and Methods

### Plasmid construction

TCF4 cDNA from *Mus musculus* was a kind gift from Prof. Dr. Moritz J. Rossner and dr Magdalena Brzózka (Ludwig-Maximilians University in Munich, Clinic of Psychiatry, Department of Molecular Neurobiology, Germany). The full-length cDNA of the canonical B+ isoform of TCF4 (UniProtKB - Q60722), with encoding amino acid residues 1-670, was amplified by PCR and cloned into the *Eco*RI and *Sal*I restriction sites of the MCS of the pEYFP-C1 vector (Clontech). Deletion mutants of TCF4 were similarly cloned into the pEYFP-C1 vector. The bHLH factor TCF4 should not be confused with the transcription factor 7-like 2 (TCF7L2; Gene 6934), which is also known as TCF4 (T-cell specific factor 4). The point mutants YFP-TCF4/550-601/L596A/L598A, YFP-TCF4/602-670/L607A/L608A, and YFP-TCF4/550-670//K606A were obtained by PCR-Mediated Site-Directed Mutagenesis, as described by Ko and Ma^64^, and cloned with *Lgu*I, *Eco*RI and *Sal*I restriction enzymes. All constructs were verified by DNA sequencing.

### Cell culture and DNA transfection

As described previously^65^ African green monkey kidney fibroblasts COS-7 (ATCC CRL-1651) and Mouse Albino neuroblastoma Neuro 2a (ECACC; Sigma-Aldrich) were cultured in Dulbecco’s modified Eagle medium (DMEM) with 4.5 g/l glucose and L-glutamine (Lonza). The culture medium was supplemented with 10% foetal calf serum (FCS). Cells were cultured at 37 °C in a 95% air/5% CO_2_ atmosphere. The cells were transfected with 3 μg of DNA/150,000 cells using Xfect Transfection Reagent (Takara Clontech) according to the manufacturer’s instructions. LMB (Sigma) was dissolved in 70% methanol at a concentration of 10^−3^ M and added to the medium during transfection at a final concentration of 10^−6^ M.

### Electrophoresis and Western blot analysis

To confirm the expression of TCF4 and TCF4 mutants in cultured COS-7 cells, additionally to the microscopy experiments, SDS–PAGE and Western blot analysis were performed. Samples of the total protein extract were prepared by replacing the cell medium 24 h after transfection (after washing cells with PBS) with a 2x SDS gel-loading buffer^66^, transferring extracts to Eppendorf tubes, boiling the samples for 5 min and then centrifuging the samples for 5 min (13,000 g). Proteins were separated by 10% SDS–PAGE and transferred to a Whatman PROTRAN Nitrocellulose Transfer Membrane (PROTRAN BA85, Schleicher and Schuell Pure, Sigma-Aldrich) with mini Trans-Blot apparatus (Bio-Rad). The membrane was blocked at room temperature (RT) for 1 h in 3% milk blocking buffer (milk powder; Milchpulver, blotting grade, Roth) that was prepared in PBS supplemented with 0.2% Tween 20 (Sigma). Next, the membrane was incubated overnight at 4 °C with anti-GFP polyclonal antibodies (Clontech) (diluted 1:300 with milk buffer), which cross-reacted with YFP. Secondary goat anti-rabbit antibodies coupled to horseradish peroxidase (Vector Laboratories) were added (1:10,000 with milk buffer) and incubated at RT for 2 h. Blots were developed using Pierce ECL Plus Western Blotting Substrate (Thermo Scientific) according to the manufacturer’s manual.

### Confocal and fluorescence microscopy

As described previously^46,67^, prior to experiments of confocal microscopy imaging, cells were seeded onto 0.17-mm-thick round glass coverslips (Menzel) and submerged in a culture medium in 2-cm diameter Petri dishes. Next, 24 h, or in some mentioned cases 48 h after transfection, the coverslips with cell cultures were transferred onto a steel holder and mounted on a microscope stage. The standard culture medium was replaced with 1 ml of DMEM/F12 without phenol red, buffered with 15 mM HEPES (Sigma) and supplemented with 2% FBS (Sigma). Next, 0.1 μl of Draq5 DNA Dye (BioStatus) was added to the cells for DNA visualization. During microscopy observation, the cell culture temperature was stabilized at 37 °C using a microincubator (Life Imaging Services Box & Cube). Images of the fluorescently labelled proteins were acquired using a Leica TCS SP5 II confocal system equipped with argon and helium neon lasers and a 63x oil objective lens (NA: 1.4). YFP was excited using 514 nm light and the emitted fluorescence was observed at a range of 525-600 nm. The excitation of Draq5 was 633 nm and emission was 650–800 nm. Gamma correction of 0.6 was set for the Draq-5 channel. At least 50 cells were observed for each cDNA construct in one experiment and we calculated the cell percentages by dividing number of cells presenting exact pattern of localization to the total number of observed cells multiplying it by 100%. At least three independent experiments were performed for each cDNA construct. Images are presented for representative cells with a typical phenotype, which is characteristic for more than 95% of cases. In the case of cells presenting many phenotypes, representative images of all the cell phenotypes are presented. Ratios between mean fluorescence intensity in cytoplasmic, nuclear and nucleolar compartments were estimated by measuring mean fluorescence intensity inside each compartment of chosen cell and presented it as an accumulated bar graph. All calculations and transformations were done using ImageJ^68^. Fluorescence microscopy was performed in 2-cm diameter Petri dishes in high glucose DMEM using an Olympus IX71 microscope with a YFP filter 24 or 48 hours after transfection.

### *In silico* analysis of the TCF4 sequence

PSIPRED (Protein Structure Prediction Server^69^; http://www.psipred.net/psiform.html) was used for predicting the secondary structure of the TCF4. The TCF4 domain architecture was predicted using SMART (Simple modular architecture tool^70^; http://smart.embl-heidelberg.de), PROSITE (a database of protein families and domains; http://expasy.org/tools/scanprosite/) and CDD (The conserved domain database^71,72^; https://www.ncbi.nlm.nih.gov/cdd/). Protein sequence analysis and prediction of the DNA binding site was performed by InterPro (a resource that provides functional analyses of proteins by classifying them into families and predicting domains and important sites^73^; https://www.ebi.ac.uk/interpro/). Sequence alignments were obtained using CLUSTAL_X^74^ (http://www.clustal.org/). Predictions for potential NLS sequences were performed using NucPred^75^, (http://www.sbc.su.se/~maccallr/nucpred/), PSORTII^76^, (http://www.psort.org/), and cNLS Mapper^77^, (http://nls-mapper.iab.keio.ac.jp/cgi-bin/NLS_Mapper_y.cgi). Predictions of potential NoLS motifs were performed using NoD (Nucleolar localization sequence Detector; http://www.compbio.dundee.ac.uk/www-nod/)^42,78^. Predictions of potential NES sequences were performed using the NetNes 1.1 server^26^, (http://www.cbs.dtu.dk/services/NetNES/), ValidNES server^79^, (http://validness.ym.edu.tw), NES Finder 0.2 (http://research.nki.nl/fornerodlab/NES-Finder.htm; and LocNES^80^ (http://prodata.swmed.edu/LocNES/LocNES.php). The NetSurfP-2.0 server was used for prediction of the relative surface accessibility of an amino acid^81^, http://www.cbs.dtu.dk/services/NetSurfP/. Predictions of protein disorder were performed using PONDR-VLXT^82,83^, http://www.pondr.com/ and MetaDisorder^84^, http://iimcb.genesilico.pl/metadisorder, while prediction of TCF4 backbone flexibility was performed with DYNAMINE^85,86^, http://dynamine.ibsquare.be/submission/. Scanning of the TCF4 sequence for Short linear motifs (SLiMs) was performed with The Eukaryotic Linear Motif (ELM) resource^87^, elm.eu.org. The TCF4-B *Mus musculus* model was generated using Phyre 2^88^ http://www.sbg.bio.ic.ac.uk/phyre2. Only 113 residues (17% of protein), encompassing mainly the bHLH domain (aa567-620) of TCF4, were modelled with a confidence higher than 90% using the following templates: c2mh0A, c5c3lA, c2ql2A and c2ypbB. The remaining 601 residues (89%) of TCF4, lacking any template and predicted to be disordered, were modelled by *ab initio*, which was highly unreliable. Result of TCF4 3D structure prediction was visualized with PyMOL (The PyMOL Molecular Graphics System, Version 1.2r3pre, Schrödinger, LLC.). Predictions of phosphorylation sites were performed using the NetPhos 3.1 server^89^, http://www.cbs.dtu.dk/services/NetPhos/ and Disorder Enhanced Phosphorylation Predictor (DEPP)^90,91^, http://www.pondr.com/cgi-bin/depp.cgi. 14-3-3-binding sites in the TCF4 were predicted using the 14-3-3-Pred server, combining predictions from three different classifiers: ANN, PSSM and SVM^92^.

## Supplementary information

Supplementary Figures
