# Supplementary material for: The subcellular localization of bHLH transcription factor TCF4 is mediated by multiple nuclear localization and nuclear export signals
Source: Sci Rep. 2019 Oct 30;9:15629. doi: 10.1038/s41598-019-52239-w (PMC6821749; doi:10.1038/s41598-019-52239-w)
Supplement: Supplementary file 1 — Supplementary Figures [file 41598_2019_52239_MOESM1_ESM.pdf]

The subcellular localization of bHLH transcription factor TCF4 is mediated by multiple nuclear localization and nuclear export signals

Beata Greb-Markiewicz, Wioletta Kazana, Mirosław Zarębski, Andrzej Ożyhar

Supporting Information:

FIGURE S1. Western blot analysis of expression of YFP tagged TCF4 and TCF4 derivatives in transfected COS-7 cells. Detection with anti-GFP antibodies. On the bottom of images presented theoretical molecular mass of proteins.

FIGURE S2. Subcellular distribution of selected TCF4 derivatives. Subcellular localization of the expressed proteins were analysed by confocal microscopy in COS-7 or N2a cells 24 h after transfection. Draq5 was added to the cells for DNA visualization. Not typical (rare events) images of subcellular distribution are presented for YFP-TCF4, YFP-TCF4/147-300, YFP-TCF4/550-670, YFP-TCF4/550-670/K606A, YFP-TCF4/1-549.

FIGURE S3. (A) Prosite prediction of domains in TCF4 protein. (B) Scansite prediction of surface accessibility. (C) PONDR, METADISORDER and DYNAMINE *in silico* analyses of the disorder character of TCF4.

FIGURE S4. Subcellular distribution of the YFP-TCF4/1-601 derivative. (A) Schematic representation of TCF4 protein. Region of the studied bHLH domain of TCF4 is shown by the red rectangle. Expressed deletion mutants of TCF4 are depicted as arrows. (B) Subcellular localization of the expressed proteins were analysed by confocal microscopy in COS-7 cells 24 h after transfection. Not typical (rare events) images of subcellular distribution are presented. (C) Subcellular localization of the expressed proteins were analysed by fluorescence microscopy in COS-7 and N2a cells 24 h after transfection in the absence or presence of additional factors such as LMB or methanol. Representative images of subcellular distribution are presented.

FIGURE S5. (A) Phosphorylation sites of the bHLH domain predicted by NetPhos and DEPP servers. (B) Results of *in silico* analyses of the bHLH domain of TCF4 by PONDR, METADISORDER and DYNAMINE servers.

Figure S1

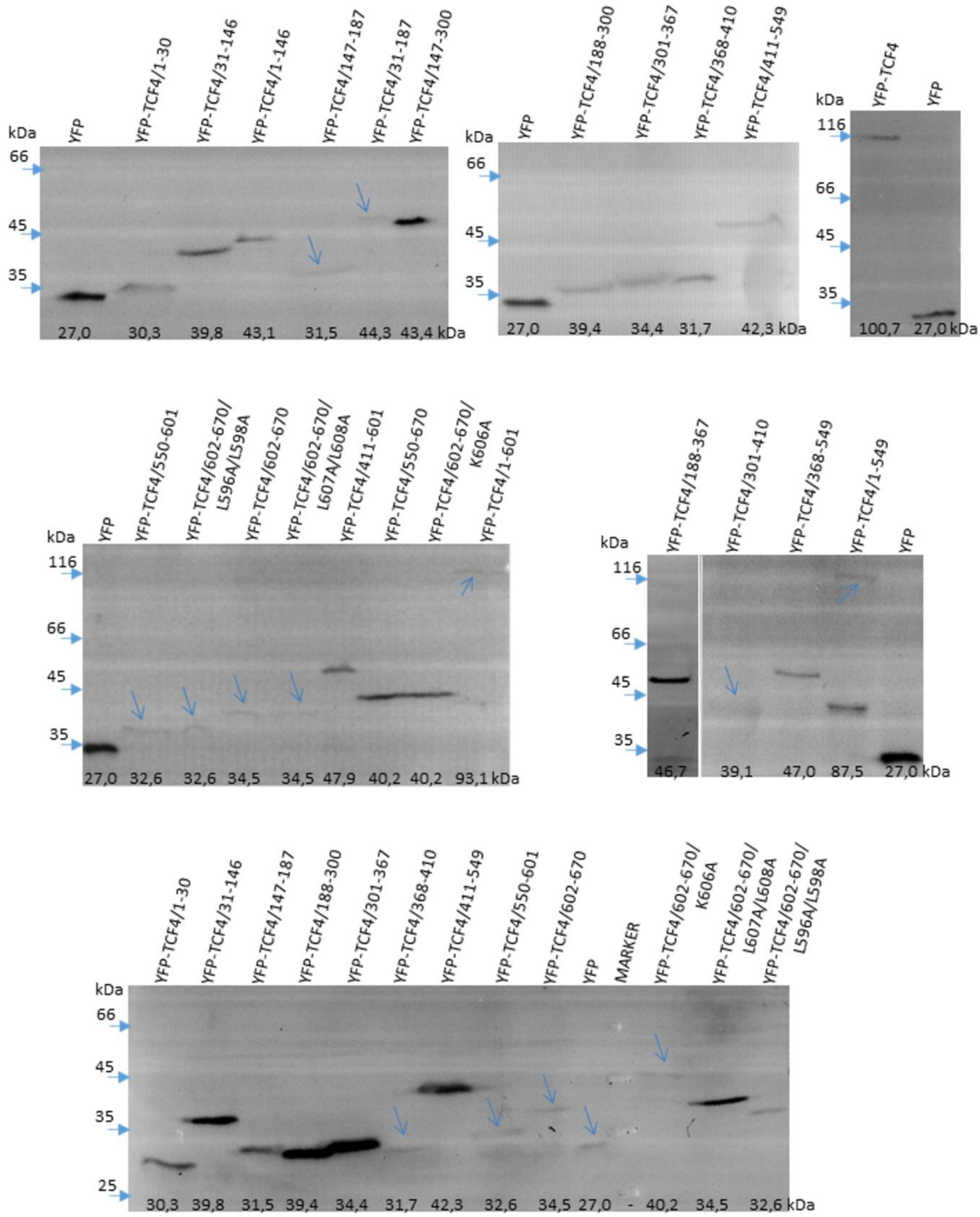
kDa
YFP
YFP-TCF4/1-30
YFP-TCF4/31-146
YFP-TCF4/1-146
YFP-TCF4/147-187
YFP-TCF4/31-187
YFP-TCF4/147-300
66
45
35
27,0 30,3 39,8 43,1 31,5 44,3 43,4 kDa
kDa
YFP
YFP-TCF4/188-300
YFP-TCF4/301-367
YFP-TCF4/368-410
YFP-TCF4/411-549
66
45
35
27,0 39,4 34,4 31,7 42,3 kDa
kDa
YFP-TCF4
YFP
116
66
45
35
100,7 27,0 kDa
kDa
YFP
YFP-TCF4/550-601
YFP-TCF4/602-670/ L596A/L598A
YFP-TCF4/602-670
YFP-TCF4/602-670/ L607A/L608A
YFP-TCF4/411-601
YFP-TCF4/550-670
YFP-TCF4/602-670/ K606A
YFP-TCF4/1-601
116
66
45
35
27,0 32,6 32,6 34,5 34,5 47,9 40,2 40,2 93,1 kDa
kDa
YFP-TCF4/188-367
YFP-TCF4/301-410
YFP-TCF4/368-549
YFP-TCF4/1-549
YFP
116
66
45
35
46,7 39,1 47,0 87,5 27,0 kDa
kDa
YFP-TCF4/1-30
YFP-TCF4/31-146
YFP-TCF4/147-187
YFP-TCF4/188-300
YFP-TCF4/301-367
YFP-TCF4/368-410
YFP-TCF4/411-549
YFP-TCF4/550-601
YFP-TCF4/602-670
YFP
MARKER
YFP-TCF4/602-670/ K606A
YFP-TCF4/602-670/ L607A/L608A
YFP-TCF4/602-670/ L596A/L598A
66
45
35
25
30,3 39,8 31,5 39,4 34,4 31,7 42,3 32,6 34,5 27,0 - 40,2 34,5 32,6 kDa

Figure S2

YFP-TCF4 in COS-7 cells (Supplement to Fig.1)

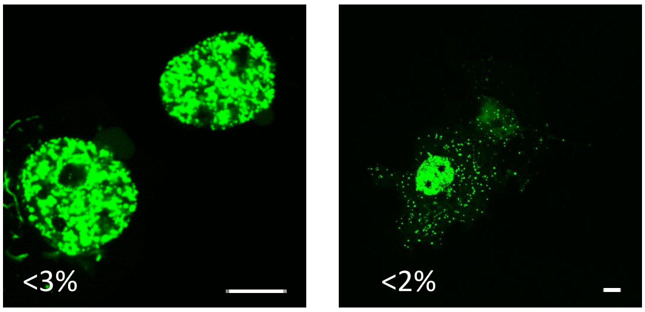

YFP-TCF4/147-300 in COS-7 cells (Supplement to Fig. 3)

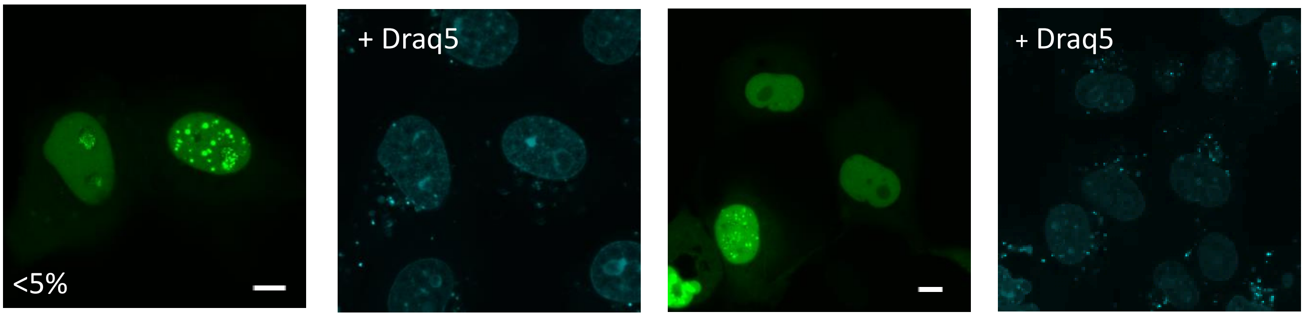

YFP-TCF4/550-670 after 24h (Supplement to Fig.8)

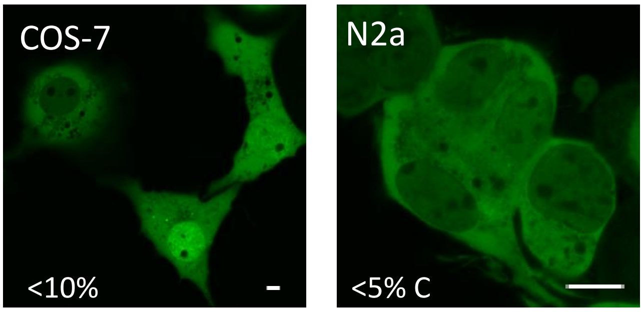

YFP-TCF4/550-670/K606A (Supplement to Fig.8)

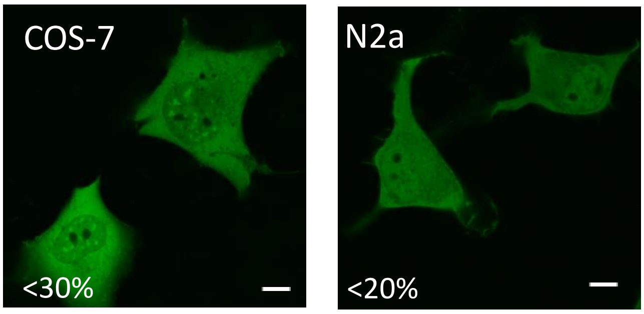

YFP-TCF4/1-549 (Supplement to Fig. 9)

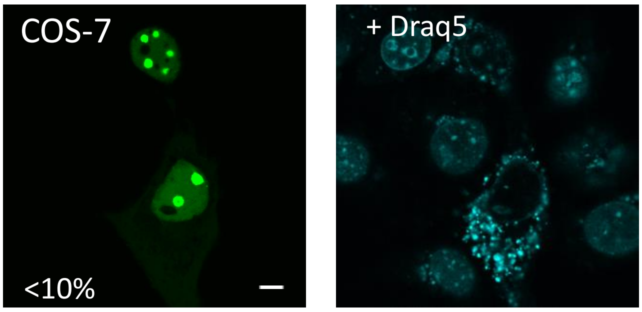

Figure S3

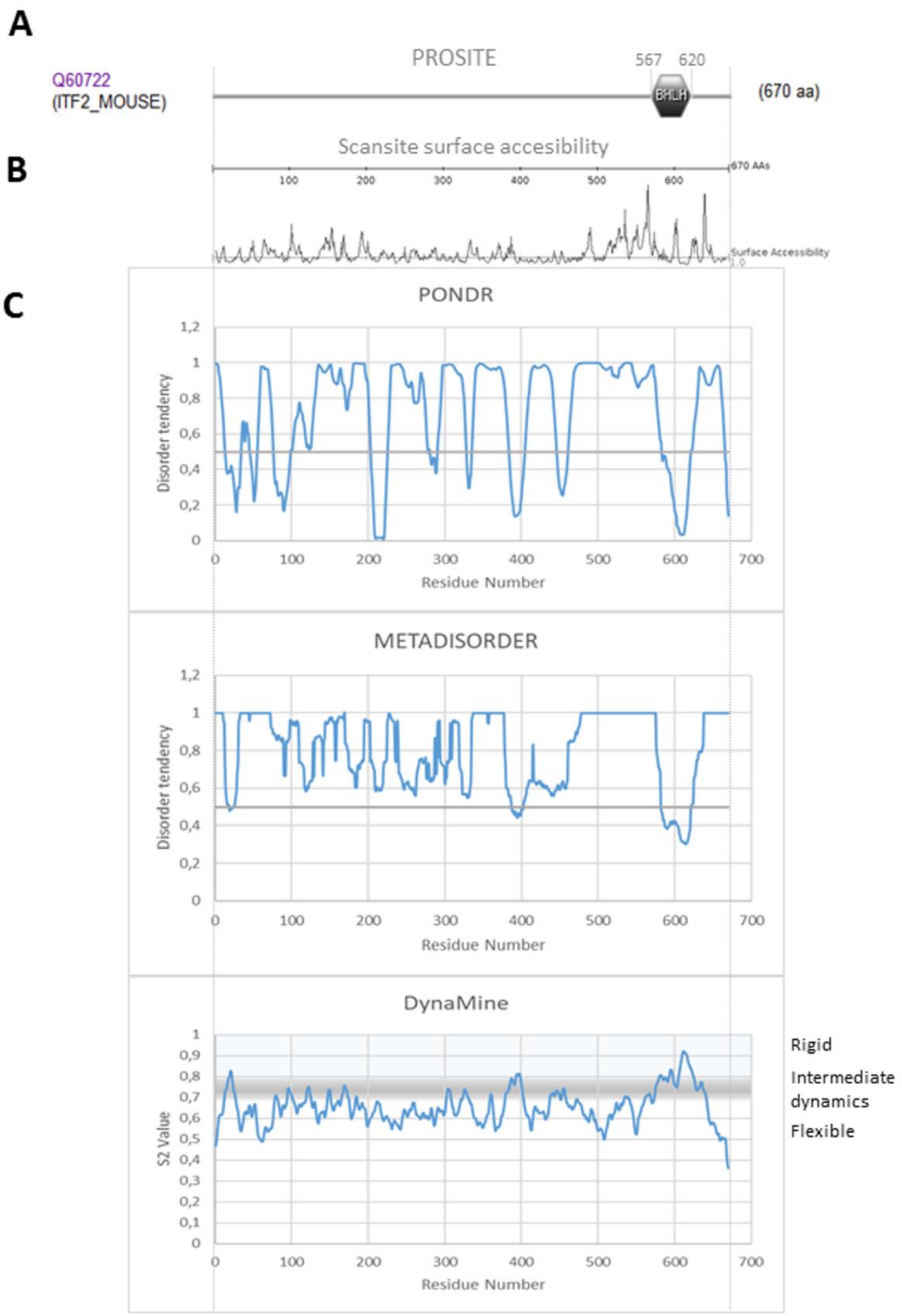
A
PROSITE
567 620
Q60722
(ITF2_MOUSE)
BHLH
(670 aa)
B
Scansite surface accesibility
670 AAs
Surface Accessibility
C
PONDR
Disorder tendency
Residue Number
METADISORDER
Disorder tendency
Residue Number
DynaMine
S2 Value
Residue Number
Rigid
Intermediate
dynamics
Flexible

Figure S4

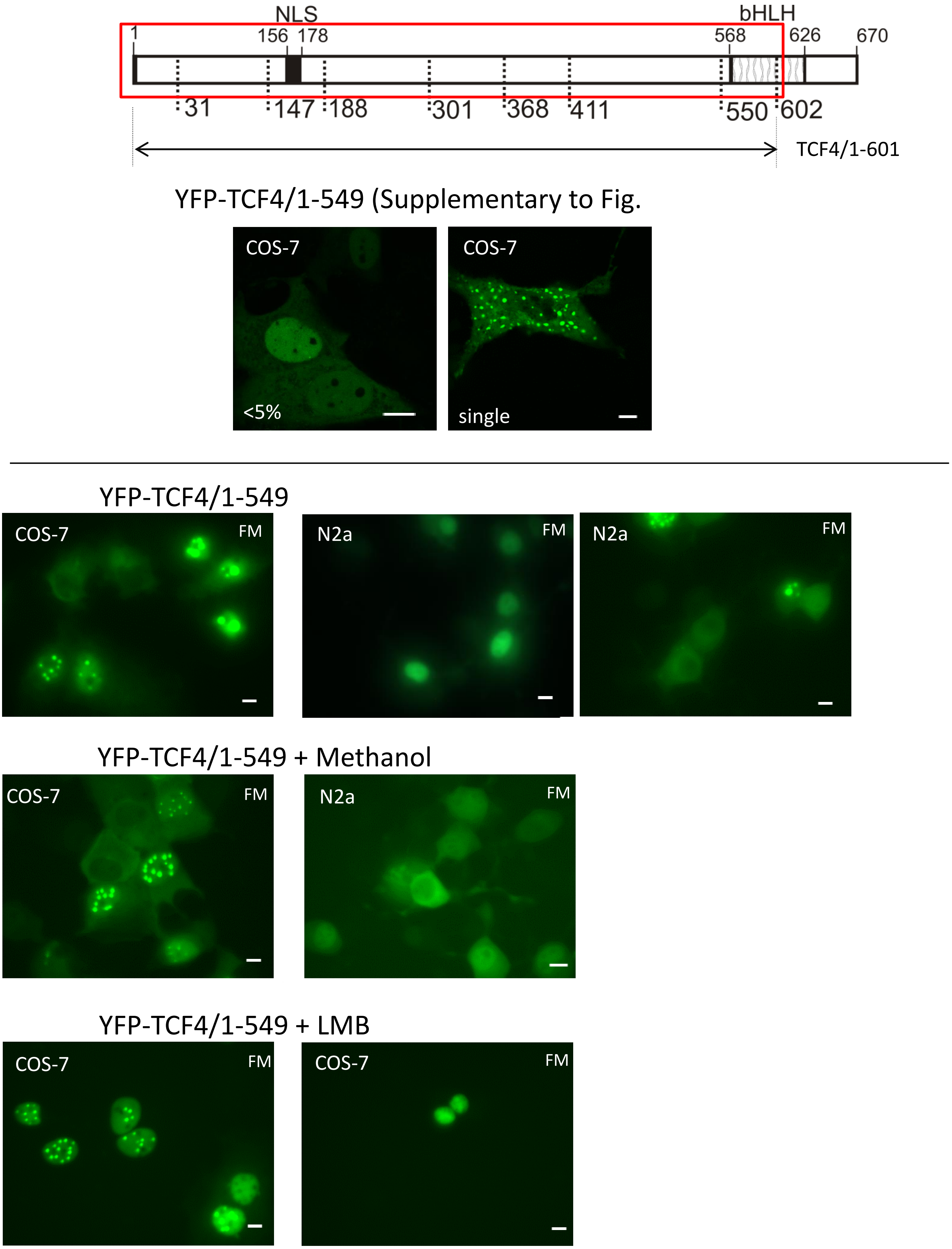
NLS
bHLH
1
156
178
568
626
670
31
147
188
301
368
411
550
602
TCF4/1-601
YFP-TCF4/1-549 (Supplementary to Fig.
COS-7
<5%
COS-7
single
YFP-TCF4/1-549
COS-7
FM
N2a
FM
N2a
FM
YFP-TCF4/1-549 + Methanol
COS-7
FM
N2a
FM
YFP-TCF4/1-549 + LMB
COS-7
FM
COS-7
FM

Figure S5

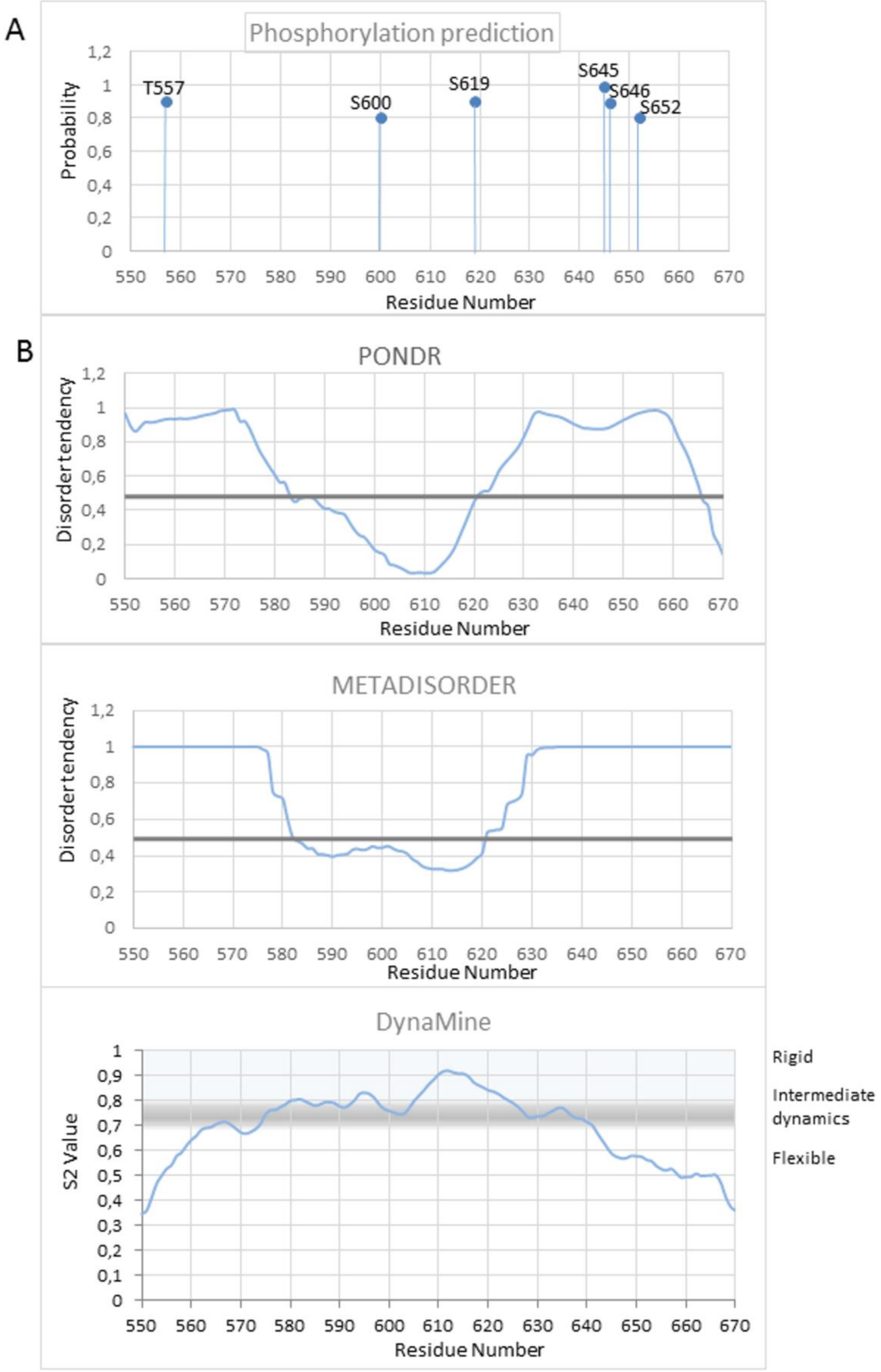
A
Phosphorylation prediction
Probability
T557
S600
S619
S645
S646
S652
Residue Number
B
PONDR
Disorder tendency
Residue Number
METADISORDER
Disorder tendency
Residue Number
DynaMine
S2 Value
Rigid
Intermediate dynamics
Flexible
Residue Number
